# A novel condition of mild electrical stimulation exerts immunosuppression via hydrogen peroxide production that controls multiple signaling pathway

**DOI:** 10.1371/journal.pone.0234867

**Published:** 2020-06-22

**Authors:** Mariam Piruzyan, Ihori Shitanda, Yuichiro Shimauchi, Go Okita, Yu Tsurekawa, Masataka Moriuchi, Yoshio Nakano, Keisuke Teramoto, Mary Ann Suico, Tsuyoshi Shuto, Hirofumi Kai

**Affiliations:** 1 Department of Molecular Medicine, Graduate School of Pharmaceutical Sciences, Kumamoto University, Kumamoto, Japan; 2 Program for Leading Graduate Schools “HIGO (Health life science: Interdisciplinary and Global Oriented) Program”, Kumamoto University, Kumamoto, Japan; National Institutes of Health, UNITED STATES

## Abstract

Different modes of exogenous electrical stimulation at physiological strength has been applied to various diseases. Previously, we extensively demonstrated the usability of mild electrical stimulation (MES) with low frequency pulse current at 55 pulses per second (MES_55_) for several disease conditions. Here we found that MES with high frequency pulse-current (5500 pulse per second; MES_5500_) suppressed the overproduction of pro-inflammatory cytokines induced by phorbol myristate acetate/ionomycin in Jurkat T cells and primary splenocytes. MES_5500_ also suppressed the overproduction of inflammatory cytokines, improved liver damage and reduced mouse spleen enlargement in concanavalin-A-treated BALB/c mice. The molecular mechanism underlying these effects included the ability of MES_5500_ to induce modest amount of hydrogen peroxide and control multiple signaling pathways important for immune regulation, such as NF-κB, NFAT and NRF2. In the treatment of various inflammatory and immune-related diseases, suppression of excessive inflammatory cytokines is key, but because immunosuppressive drugs used in the clinical setting have serious side effects, development of safer methods of inhibiting cytokines is required. Our finding provides evidence that physical medicine in the form of MES_5500_ may be considered as a novel therapeutic tool or as adjunctive therapy for inflammatory and immune-related diseases.

## Introduction

Electrical stimulation is a versatile treatment modality that has gained increasing attention, and is one of the oldest and most effective modalities used in physical medicine [1]. It is applied in various medical fields, such as wound healing [2], nerve repair [3] and muscular dystrophy recovery [4], among others. Although little is known about the cellular and molecular mechanisms of the effects of electrical stimulation, our work and others’ have gradually shed light on that aspect [5–7]. We have previously characterized mild electrical stimulation (MES) as a treatment approach, and optimized its biological activities [8, 9]. MES has no toxicity and does not induce muscle contraction [6, 10]. We have shown that combined treatment of MES and heat shock (HS) improved insulin resistance in mice [11], reduced adiposity and hyperglycemia in humans [12, 13], ameliorated hepatic ischemia-reperfusion injury in mice [14], declined the progressive nephritis in murine model of Alport syndrome [15] and decreased the inflammation in imiquimod-induced psoriasis mouse model [9]. In the case of the effect of MES on limiting insulin resistance and decreasing adiposity, mechanistically, MES activated the phosphoinositide 3-kinase (PI3K)-Akt signaling pathway, leading to enhanced insulin signaling and improved metabolism [11, 12]. A common factor that we observed in the effect of MES on ameliorating the diabetic phenotype, ischemia, nephritis and psoriasis is the reduction of the levels of pro-inflammatory cytokines. This was notable because an inflammatory milieu is a causative and/or exacerbating factor in these diseases. Based on our previous findings, we postulated that MES has an immunomodulatory effect, and if so, could be used to modulate inflammatory conditions.

Inflammation is a protective response of host cells against infection, stress and injury that is normally controlled and self-limited [16]. Failure to dampen the signaling pathway of inflammation leads to pathologic or chronic inflammation. Moreover, inappropriate inflammatory response when there are no foreign substances to fight off leads to autoimmunity. Lymphocytes from the T helper cell lineage have an important role in the onset and maintenance of autoimmune inflammatory processes and in chronic inflammation [17]. When activated, T cells produce inflammatory mediators or cytokines [18]. Among such cytokines, interleukin (IL)-2 plays a crucial role in the generation and maintenance of regulatory T cells against autoimmune diseases [19]. However, the excessive production of IL-2 and other cytokines can lead to inflammatory diseases. Therefore, suppressing excessive production of inflammatory cytokines is effective in the treatment of various inflammation-related diseases. In controlling excessive inflammation, immune-modulatory drugs such as cyclosporine (CsA) are widely used. Mechanistically, CsA inhibits calcineurin which leads to the dephosphorylation and impaired nuclear translocation of nuclear factor of activated T cells (NFAT) [20]. NFAT regulates the transcription of IL-2 and consequently, T cell activation [21]. Calcineurin inhibitors are being used in many immune-related diseases, such as rheumatoid arthritis (RA) [22] and atopic dermatitis [23]. Most notably, they are used as immunosuppressants for organ transplantation with excellent short outcome. However, CsA and similar drugs induce long-term toxicity such as renal dysfunction [24] and hypertension [25], necessitating a search for non-toxic alternatives for longer term use.

Because MES has been shown to be non-toxic, and may have inflammation-dampening effect [12, 13], we further explored the effect and mechanism of MES on inflammatory response and immune reaction. MES at 5500 pulses per second (pps) suppressed acute inflammation in immune-stimulatory conditions, and the mechanism underlying this process was the ability of MES to affect immune-related pathways *via* the involvement of hydrogen peroxide (H_2_O_2_) production. The present study contributes to our knowledge on the effects and mechanisms of MES that could lead to the adjunctive use of MES with existing drugs for excessive inflammation and autoimmune diseases.

## Materials and methods

### Cell culture and PMA/Io treatment

Human Jurkat T cell line was obtained from the American Type Culture Collection (ATCC) and used to study the effect of MES on immune system. Cells were cultured in RPMI-1640 medium (Wako) supplemented with 10% inactivated fetal bovine serum (FBS), 1% antibiotics (P/S; Penicillin G (100 units/mL), Streptomycin (100 μg/mL)). Inactivation of FBS was performed in 56°C for 30 min. The culture was not supplemented with growth factors. Cells were maintained (not more than 3 x 10^6^ cells/mL) at 37°C in humidified 5% CO_2_ incubator. During treatment with drugs or MES, FBS-free medium was used. For stimulation, cells were treated with 10 nM phorbol 12-myristate 13-acetate and 500 nM Ionomycin (PMA/Io), and incubated for 3 hr at 37°C. Medium was changed and total RNA was isolated.

### Experimental animal

Six-week-old or thirteen-week-old female BALB/c mice were obtained from Charles River Laboratories, Inc. (Kanagawa, Japan). Mice were housed in a vivarium in accordance with the guidelines of the animal facility center of Kumamoto University. Mice were fed with food and water *ad libitum*. All the animal experiments were approved by the Animal Care and Use Committee of Kumamoto University (C28-068). All methods were performed in accordance with the relevant guidelines and regulations.

### ConA-induced mouse model for inflammation

For inflammation model, we used intravenous injection of the plant lectin concanavalin A (ConA), which stimulates TCR and specifically activates T cells. ConA is widely used for acute immune-mediated hepatitis in mice. By accumulating into inner organs, such as liver and spleen, ConA induces acute inflammation. We pre-treated 7-week-old BALB/c mice with MES_5500_ (4 V/cm) for 20 min. One mg/kg ConA was injected through the tail vein of BALB/c mice after the first MES_5500_ treatment. Mice were treated again with MES_5500_. Four or eight hours after the last MES_5500_ treatment, mice were anesthetized using isoflurane and sacrificed by cervical dislocation. Total RNA was extracted from mice spleen and liver, and subjected to quantitative real-time RT-PCR to detect the mRNA level of inflammatory cytokines. For CsA treatment, mice were subcutaneously injected twice with CsA (3.25 mg/kg) at 15 hr and at 40 min before ConA injection.

### Primary splenocyte isolation from mouse spleen

Primary splenocytes were prepared by aseptically removing the spleens from the BALB/c mice. The spleens were homogenized in RPMI medium (with inactivated FBS) using a syringe piston. Single spleen cells were collected and treated by lysing the red blood cells with ACK lysis buffer (Gibco® by Life Technologies). Splenocytes were adjusted to a concentration of 1 × 10^7^ cells/ml in RPMI medium with a hemocytometer using the trypan blue dye exclusion method. Splenocytes were plated in 6-well plates. The plates were incubated at 37°C in a humidified incubator with 5% CO_2_ for up to 24 h. Splenocytes were treated with MES_5500_ for 10 min, and medium was changed. Cells were stimulated with PMA/Io for 3 hr, then collected for analysis.

### *In vitro* MES treatment

Jurkat T cells were plated on 6-well culture dishes and were treated with MES_5500_. Electrodes connected to multifunction generator (#WF1973; NF Corporation, Japan) were put into the culture media and MES_5500_ stimulation was delivered using 2 V/cm (5500 pps) of pulse-current with individual pulse duration of 0.1 ms. The culture plate with electrodes was immersed in a water bath at 37°C. Treatment was performed for 10 min. Treatment of cells with MES_55_ was similar to MES_5500_ except that MES_55_ stimulation has pulse duration of 55 pps as described previously [7].

### *In vivo* MES treatment

Seven-week-old BALB/c mice were treated with MES_5500_ before and after a single i.v. injection of ConA. For MES_5500_ treatment, mouse was placed in a well-ventilated 12 cm x 10 cm (width x height) chamber, which was designed for the animal treatment as described previously [11]. Electrical stimulation was delivered to un-anesthetized test animal through a pair of 10-cm diameter electro-conductive rubber electrodes, which were padded with moist soft cotton cloth. The electrodes can be adjusted to allow contact with the animal (S2A Fig). The electrodes are connected to a multifunction generator that delivered 8 V (4 V/cm, 5500 pps) of pulse current with pulse duration of 0.1 ms (S2B Fig). For the control group, mice were sham treated by putting them in the chamber with similar set-up as above but without conducting MES_5500_.

### RNA isolation from cells

Jurkat T cells were plated on 6-well plates at 3~5 x 10^6^ cells. Cells were treated with MES (5500 pps, 0.1 msec, 2V/cm) for 10 min at 37°C. Medium was changed and cells were stimulated with T cell activator PMA/Io. After 3 hr, total RNA extraction was performed by using RNAiso plus (Takara). Total RNA was measured with Epoch Spectrophotometer (Bio-Tek). High purity RNA with OD_260_/OD_280_ more than 1.8 was obtained.

### RNA isolation from mouse spleen and kidney

Mice were anesthetized and sacrificed 4 or 8 hr after the last MES_5500_ treatment. Spleen and liver were surgically collected immediately after sacrifice and rotated for 12 hr at 4°C in RNAlater solution (Ambion). Organs were transferred to fresh tubes and frozen in -80°C. Organs were cut into small pieces with surgical scissors and homogenized, and total RNA was collected using RNAiso plus. Total RNA was measured with Epoch Spectrophotometer (Bio-Tek). High purity RNA with OD_260_/OD_280_ more than 1.8 was obtained.

### Real-time quantitative RT-PCR

Real-time quantitative RT-PCR analyses were carried out with SYBR Ex Taq^TM^ (Takara Bio Inc.) as we reported previously [26]. The threshold cycle values for each gene amplification were normalized by subtracting the threshold cycle value calculated for GAPDH or 18s rRNA (internal control). The normalized gene expression values are expressed as the relative quantity of gene-specific mRNA. The sequences of primers used for quantitative PCR are listed in Table 1. PCR results were quantified with Livak’s 2^-ΔΔC^_T_ method [27].

**Table 1:** Primers for RT-PCR.

Gene	Primer sequence	
	Sense	Antisense
Human IL-2	5’-GGATGCAACTCCTGTCTTGC-3’	5’-GTGGCCTTCTTGGGCATGTA-3’
Human HO-1	5’-GGGAATTCTCTTGGCTGGCT-3’	5’-GCTGCCACATTAGGGTGTCT-3’
Human IL-8	5’-CTGGCCGTGGCTCTCTTGT-3’	5’-CCTTGGCAAAACTGCACCTT-3’
Human IL-1β	5’-ACGAATCTCCGACCACCACT-3’	5’-CCATGGCCACAACAACTGAC-3’
Human IL-10	5’-AGAACCTGAAGACCCTCAGGC-3’	5’-CCACGGCCTTGCTCTTGTT-3’
Human TGF-β	5’-ACCTGCCACAGATCCCCTAT-3’	5’-CTCCCGGCAAAAGGTAGGAG-3’
Human TNF-α	5’-CAGCCTCTTCTCCTTCCTGA-3’	5’-TGAGGTACAGACCCTCTGAT-3’
Mouse IL-2	5’-CCTGAGCAGGATGGAGAATTACA-3’	5’-TCCAGAACATGCCGCAGAG-3’
Mouse IFN-γ	5’-CAACAACATAAGCGTCATTGAATCACAC-3’	5’-GTTGACCTCAAACTTGGCAATACTC-3’
Mouse TNF-α	5’-CATCTTCTCAAAATTCGAGTGACAA-3’	5’-TGGGAGTAGACAAGGTACAACCC-3’
Mouse IL-6	5’-GAGGATACCACTCCCAACAGACC-3’	5’-AAGTGCATCATCGTTGTTCATACA-3’
Mouse IL-8	5’-TGTCAGTGCCTGCAGACCAT-3’	5’-CCTCGCGACCATTCTTGAGT-3’
Mouse IL-10	5’-ATCGATTTCTCCCCTGTGAA-3’	5’-TTCATGGCCTTGTAGACACCT-3’
Mouse TGF-β	5’-CACCTGCAAGACCATCGACAT-3’	5’-GAGCCCTTAGTTTGGACAGCATCTG-3’

### Protein isolation from cells

Jurkat T cells were plated on 6-well plates at 3~5 x 10^6^ cells. Cells were treated with MES (5500 pps, 0.1 msec, 2V/cm) for 10 min at 37°C. Medium was changed and cells were stimulated with T cell-activating PMA/Io. After 1 or 3 hr, proteins were isolated. Floating cells were centrifuged (4°C, 12000 rpm, 2 min), culture medium was removed and the pellet was washed with phosphate buffered saline (PBS). Cell pellets were resuspended in signal detection buffer (25 mM HEPES, 10 mM Na_4_P_2_O_7_・10H_2_O, 100 mM NaF, 5 mM EDTA, 2 mM Na_3_VO_4_, 1% TritonX-100, MQ) containing 1% v/v protease inhibitor cocktail and Na_3_VO_4_, and rotated for 2 hr at 4°C. Cells were centrifuged (4°C, 12000 rpm, 15 min) and protein lysates were collected. Protein quantification was performed with bicinchoninic acid (BCA) assay.

### Nuclear isolation from cells

Nuclear lysate extraction was performed as described previously [28]. PBS-washed cell pellets were resuspended by gentle pipetting in cold buffer containing 10 mM HEPES-KOH, 10 mM KCl, 0.1 mM EDTA, 0.1 mM EGTA (pH 8.0), 1mM dithiothreitol, and 0.5 mM phenylmethylsulfonyl fluoride (PMSF). The cells were then incubated on ice for 15 min, after which 10% Nonidet P-40 solution was added, and the tube was vortexed for 10 sec. The homogenate was centrifuged (15,000 rpm) for 1 min at 4°C. The nuclear pellet was resuspended in ice-cold buffer containing 20 mM HEPES-KOH, 0.4 M NaCl, 1mM EDTA, 1 mM EGTA (pH 8.0), 1 mM dithiothreitol, and 1 mM PMSF, and the tube was vortexed for 15 min at 4°C. Then the nuclear extract was centrifuged (15,000 rpm) for 5 min at 4°C, and the nuclear supernatant was collected. Protein quantification was performed with Bradford quantification assay.

### Western blotting

Protein lysates from Jurkat T cells were subjected to SDS-PAGE on 8.5 or 10% polyacrylamide gels. Proteins were electroblotted from the gels to polyvinylidene difluoride membrane (250 mA, 2 hr). The membranes were blocked in 5% skim milk and 0.1% Tween 20 in TBS for 1 hr at RT, and incubation with the primary antibodies was for 1 hr at RT or kept overnight at 4°C. The membranes were then washed three times in 0.05% Tween 20 in PBS or 0.1% Tween 20 in Tris-buffered saline (TBS) and probed with the indicated antibodies and their respective HRP-conjugated secondary antibodies for 1 or 2 hr at RT. The list of antibodies used is shown in S1 Table. The proteins were reacted with Super Signal west pico chemiluminescent substrate (PIERCE) or Amersham ECL prime and detected with LAS-4000 mini (FUJIFILM).

### Histological analysis

Liver samples were fixed in 10% paraformaldehyde and embedded in paraffin. Five-μm tissue sections were stained with haematoxylin and eosin (H&E) according to the manufacturer’s protocol. Images were acquired using Bio-Revo imaging (BX-X700; KEYENCE, Osaka, Japan). Necrotic area was characterized by occurrence of hepatic lesions and was quantified 8 hr after ConA. Necrotic areas were assessed from 10 random high-powered fields in 4 mice and calculated in percent of total area.

### Blood ALT and AST analysis

For quantification of mouse plasma/serum glutamic pyruvic transaminase (alanine aminotransferase) and glutamic oxaloacetic transaminase (aspartate aminotransferase), FUJI DRI-CHEM SLIDE GPT/ALT-PIII and FUJI DRI-CHEM SLIDE GOT/AST-PIII were used, respectively. Procedure was done according to the manufacturer’s protocol.

### H_2_O_2_ and catalase treatment

Jurkat T cells were treated with H_2_O_2_ (Abcam) at a concentration of 200 μM for 10 minutes at 37°C. For H_2_O_2_ with catalase treatment, catalase was added to H_2_O_2_ and incubated for 1 hr (200 U/ml of catalase (Wako); 1 U catalase = 1 μM H_2_O_2_/min at pH 7.0, 25°C). Then Jurkat T cells were incubated with catalase-treated H_2_O_2_ and stimulated with PMA/Io for 3 hr. For MES_5500_ treatment with catalase, cells were treated with MES_5500_ for 10 min, then added with catalase and PMA/Io, and incubated for 3 hr. For MES_CFM_ with catalase treatment, catalase was added to MES_CFM_ for 1 hr. Catalase-treated MES_CFM_ was added to cells for 10 min. and stimulated with PMA/Io.

### H_2_O_2_ quantification *in vitro*

H_2_O_2_ was measured with Hydrogen Peroxide Assay Kit (Abcam). Cells at 2 x 10^6^ were washed with cold PBS, then resuspended with 500 μl assay buffer on ice. Cells were homogenized by using a Dounce homogenizer, centrifuged, and the supernatant was transferred to a clean tube and put on ice. Deproteinization was done with cold perchloric acid (PCA), and excess PCA was precipitated with 2M KOH. After neutralization, pH was checked (pH 6.5–8) and samples were centrifuged at 13,000 x g for 15 minutes at 4°C. Supernatant was used for the assay. In the presence of Horse Radish Peroxidase (HRP), the OxiRed Probe reacts with H_2_O_2_ to produce red fluorescence (Ex/Em = 535/587).

### H_2_O_2_ quantification *in vivo*

Thirteen-week-old BALB/c mice were treated with MES_5500_ 4 V/cm (8 V) for 30 min. Mice were anesthetized with isoflurane, and blood from inferior vena cava was drawn using heparin-coated syringe. Blood serum was obtained by centrifuging at 3000 x g for 15 min in a refrigerated centrifuge. The resulting supernatant is designated as serum. Ice cold 4 M perchloric acid (PCA) was added to samples to a final concentration of 1 M and incubated on ice for 5 min. Samples were centrifuged at 13000 x g for 2 min at 4°C in a cold centrifuge and the supernatant was transferred to a fresh tube. Volume of the supernatant was measured. Excess PCA was precipitated by adding ice-cold 2M KOH that equals 34% of the supernatant, and samples were vortexed. After neutralization, pH was determined to be approximately 6.5–8. Samples were centrifuged at 13000 x g for 15 min at 4°C, and supernatant was collected and used for the assay using H_2_O_2_ Assay Kit (Abcam). In the presence of Horse Radish Peroxidase (HRP), the OxiRed Probe reacts with H_2_O_2_ to produce red fluorescence (Ex/Em = 535/587).

### Cell toxicity detection

Cell toxicity was measured with Cytotoxicity Detection Kit ^PLUS^ (LDH) (Roche). Cells were treated with MES_5500_ or 200 μM H_2_O_2_ for 10 minutes, then medium was changed. Cells were incubated at 37°C. After 24 hr of incubation, cells were centrifuged at 12000 rpm for 15 minutes and medium was collected. The cell pellet was treated with 1 ml 1% Triton-X and incubated for 30 min at 37°C. After 30 min, cells were centrifuged at 12000 rpm for 15 min and lysates were collected. Medium and lysate were diluted with FBS-free medium. Fifty μl of diluted samples was applied to 96-well assay plate. Fifty μl of lactate dehydrogenase (LDH) reagent solution was added to the samples, and incubated for 30 min at RT in the dark. Absorbance was measured (Mode; Dual, 450nm = Abs, 595nm = Reference).

### Statistical analysis

Data are presented as mean ± SD. Normal distribution was evaluated by Shapiro-Wilk tests. Normally distributed variables were compared by Student’s *t* test or one-way ANOVA with Tukey-Kramer test. Non-normally distributed variables were compared by Wilcoxon test. *P* value of <0.05 was considered statistically significant. JMP software was used for statistical analysis

## Results

### MES_5500_ inhibited PMA/Io-induced overexpression of inflammatory cytokines in Jurkat T cells and primary splenocytes

**Fig 1:**
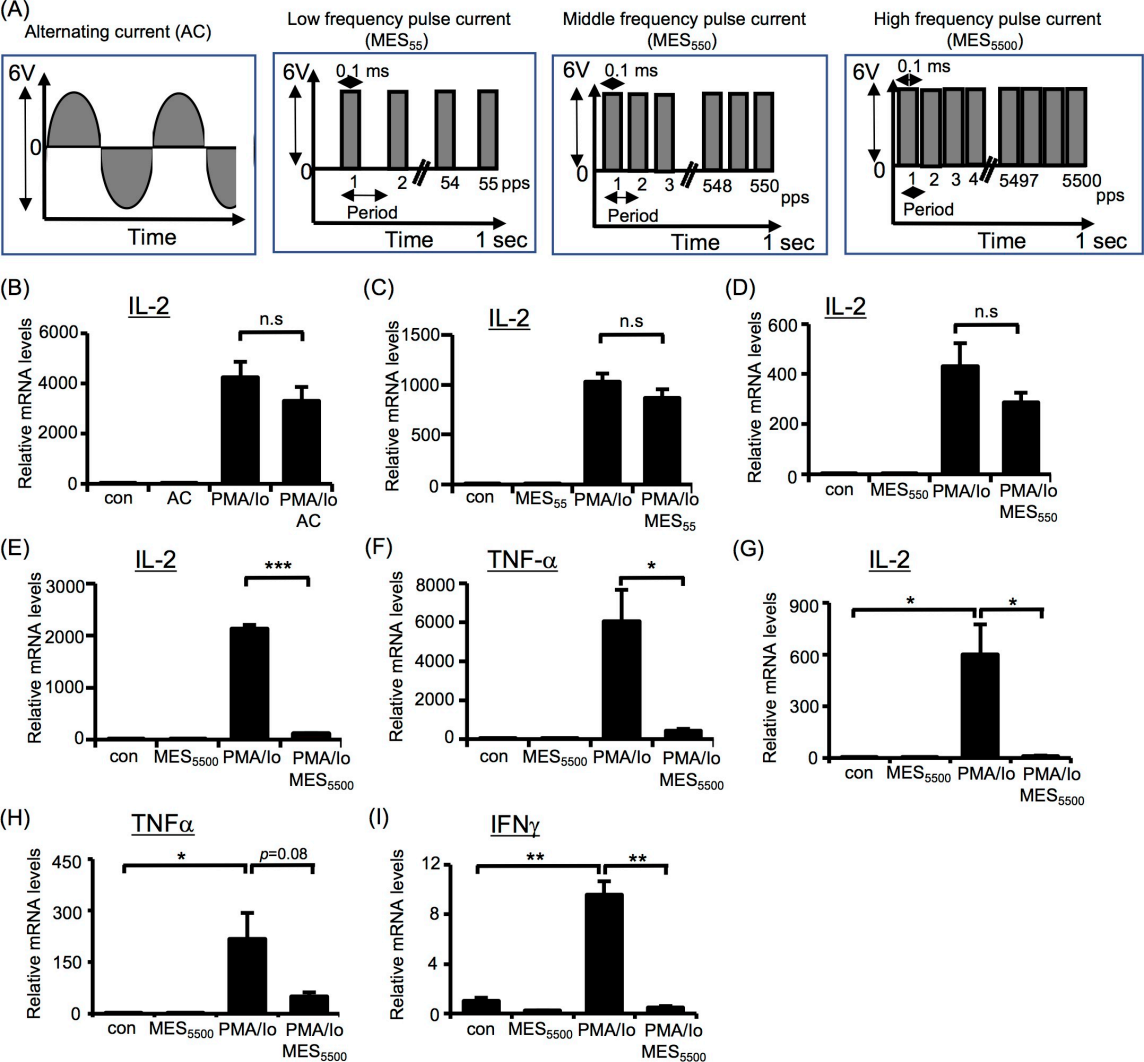
MES_5500_ is an effective immunosuppressor *in vitro*. (A) Schematic diagrams of alternating current (AC), and different frequency pulse currents (low MES_55_, middle MES_550_ and high MES_5500_). (B-F) IL-2 and TNF-α mRNA levels were analyzed in Jurkat T cells treated with AC (B), MES_55_ (C), MES_550_ (D) or MES_5500_ (E, F), and stimulated with PMA/Io. (G-I) Primary mouse splenocytes treated with MES_5500_ and stimulated with PMA/Io were analyzed by qRT-PCR to detect the indicated genes. Data were normalized to the level of GAPDH mRNA (internal control). Data are presented as mean ± S.D. (n = 3 per group). *P < 0.05, ***P<0.001 assessed by Tukey-Kramer test. (n.s.; not significant). The data shown are representative of 2 or more independent experiments.

First, we checked whether different types of electrical stimulation (Fig 1A) can suppress the overproduction of inflammatory cytokines in immune T cells. Jurkat cells were treated with alternating current (AC), or pulse current with low, middle and high frequency (MES_55_, MES_550_ or MES_5500_, respectively) for 10 min and were stimulated with PMA/Io for 3 hr. Quantitative RT-PCR analysis was performed to detect IL-2 mRNA level. None of these electrical stimulations affected the expression of IL-2 in basal state. Addition of PMA/Io highly induced IL-2 expression in Jurkat cells that was not blocked by AC, MES_55_ or MES_550_ (Fig 1B–1D). In contrast, MES_5500_ strongly suppressed the overexpression of IL-2 (Fig 1E). MES_5500_ also suppressed other PMA/Io-induced cytokines such as TNF-α (Fig 1F), IL-8, IL-4, and IL-1β (S1A–S1C Fig) in T cells. Furthermore, we isolated and cultured splenocytes from mice. These cells were treated with MES_5500_, then stimulated with PMA/Io. Consistent with the results in Jurkat cells, MES_5500_ treatment decreased the mRNA levels of PMA/Io-induced IL-2, TNF-α and IFN-γ (Fig 1G–1I) in mouse primary splenocytes. To monitor the response of T cell-specific and/or pro-inflammatory cytokine, we checked the production of IL-10, a well-known anti-inflammatory cytokine, and TGF-β. MES_5500_ increased the mRNA level of IL-10 in the presence of PMA/Io (S1D Fig) but suppressed the mRNA level of TGF-β (S1E Fig). The reason for the suppression of TGF-β by MES_5500_ is unclear. Taken together, these data indicate the characteristic anti-inflammatory action of MES_5500_ *in vitro*.

### MES_5500_ suppressed ConA-induced acute inflammation in mice

**Fig 2:**
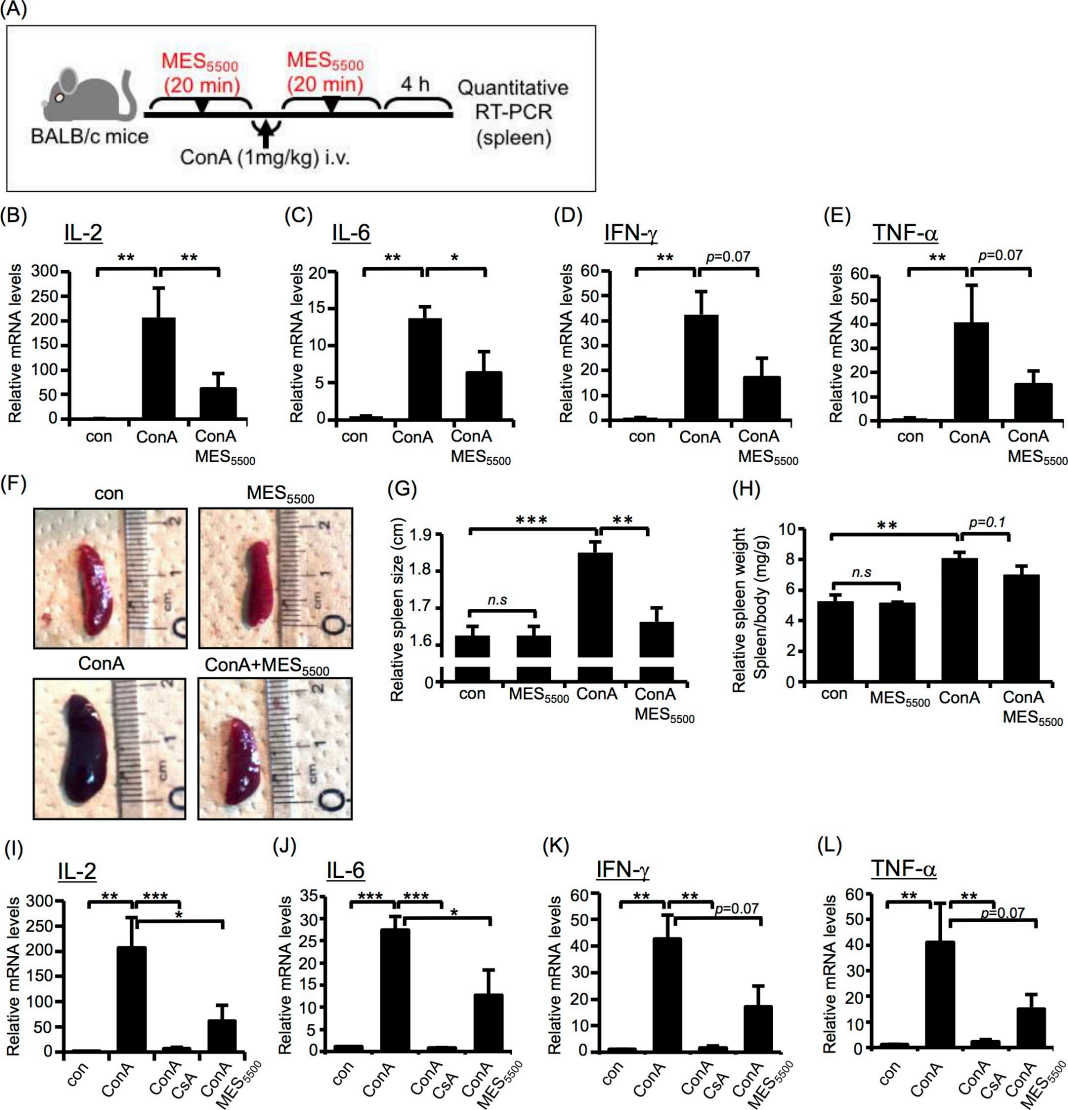
MES_5500_ suppresses overexpression of inflammatory cytokines *in vivo*. (A) Schematic diagram of the experimental protocol for *in vivo* treatment with MES_5500_. (B-H) BALB/c mice were treated with MES_5500_ for 20 min, and injected with ConA (i.v.; 1 mg/kg). Mice were treated again with MES_5500_. Four hr later, total RNA from mice spleen was extracted for analysis of the indicated cytokine (B-E). Mouse spleen size (F-G) and weight (H) are shown. (I-L) BALB/c mice were injected with CsA (see Methods), and treated with MES_5500_ and ConA as above. Total RNA from mice spleen was extracted to analyze the indicated cytokines. Values were normalized to GAPDH (internal control). Bars are mean ± S.D. (n = 8). (B, E, H, L) **P < 0.01 assessed by Tukey-Kramer test. (C, D, G, I-K) *P < 0.05, **P < 0.01, ***P<0.001 assessed by Wilcoxon test.

**Fig 3:**
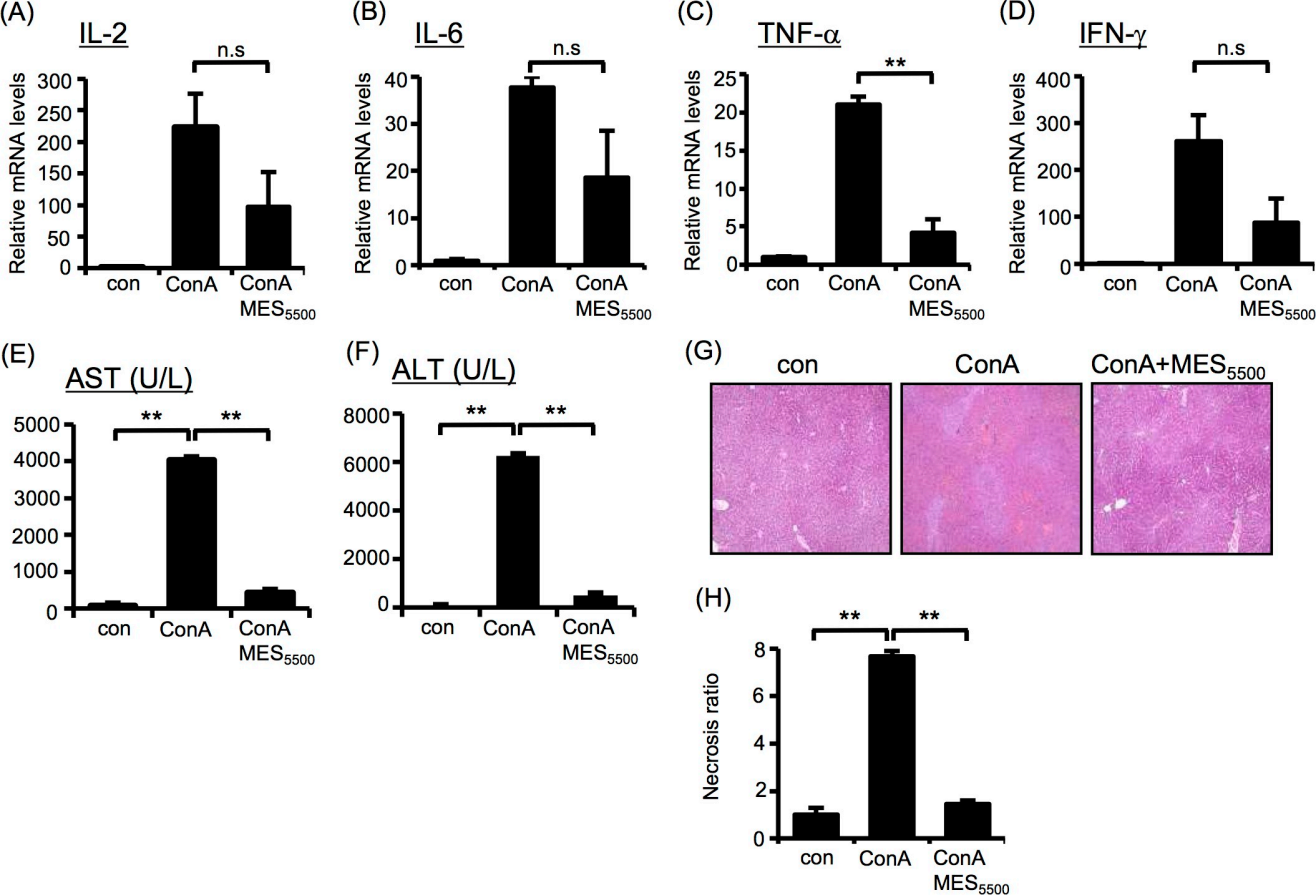
MES_5500_ improves ConA-induced hepatitis in mice. (A-G) BALB/c mice were treated with MES_5500_ and ConA as above. Total RNA from mice liver (A-D) were extracted for analysis of the indicated cytokines. AST (E) and ALT (F) levels were analyzed in mouse blood serum. (G) H&E staining of liver tissue. (H) Quantification of necrotic area of (G). (A-D) Values were normalized to GAPDH (internal control). (A-F, H) Bars are mean ± S.D. (n = 6). (A-E, H) **P < 0.01 assessed by Tukey-Kramer test. (n.s.; not significant). (F) **P < 0.01 assessed by Wilcoxon test.

Next, we investigated whether MES_5500_ can suppress inflammatory cytokine expression *in vivo*. As an inflammation model, we used ConA-induced mouse model of acute hepatitis. The schematic diagram of experimental protocol (Fig 2A), the diagram of *in vivo* MES_5500_ treatment set-up and the multifunction generator used are shown in S2A–S2B Fig. The injection of ConA increases the expression of T cell-derived IL-2, TNF-γ and other cytokines [29]. MES_5500_ treatment induced statistically significant down-regulation of overexpressed IL-2 and IL-6 (Fig 2B and 2C) and tended to reduce mRNA levels of IFN-γ and TNF-α in the spleen (Fig 2D and 2E). MES_5500_ also suppressed the ConA-induced enlargement of the spleen (Figs 2F–2G and S2C) although it did not significantly reduce the ConA-induced increase of spleen weight (Fig 2H). At the basal condition, MES_5500_ did not affect spleen size and weight (Fig 2F–2H). Next, we compared the effect of MES_5500_ with the effect of CsA, a strong immunosuppressant. MES_5500_ treatment had similar albeit milder suppressive effect on inflammatory cytokines (Fig 2I–2L). We also checked liver inflammation after 8 hr of ConA injection. Although statistical significance was not achieved, MES_5500_ suppressed the ConA-induced expression levels of inflammatory cytokines IL-2, IL-6, TNF-α and IFN-γ (Fig 3A–3D). Moreover, MES_5500_ treatment suppressed the levels of liver damage markers aspartate transaminase (AST) and alanine aminotransferase (ALT) (Fig 3E and 3F), and decreased necrosis in inflamed liver (Fig 3G and 3H) indicating that MES_5500_ treatment may ameliorate inflammatory damage and improve liver function. To further support our results using ConA, we monitored the effect of MES_5500_ on inflammatory cytokines in ConA-stimulated T cells (S3A–S3D Fig) and splenocytes (S3E–S3H Fig). MES_5500_ decreased the mRNA levels of ConA-induced IL-2, IL-8, IL-4, TNF-α and IFN-γ, and increased the mRNA level of anti-inflammatory cytokine IL-10 (S3A–S3H Fig, as indicated). Together, these data revealed that MES_5500_ suppresses inflammation *in vitro* and *in vivo*.

### MES_5500_ inhibits NF-κB and NFAT nuclear translocation and activates NRF2 pathway

**Fig 4:**
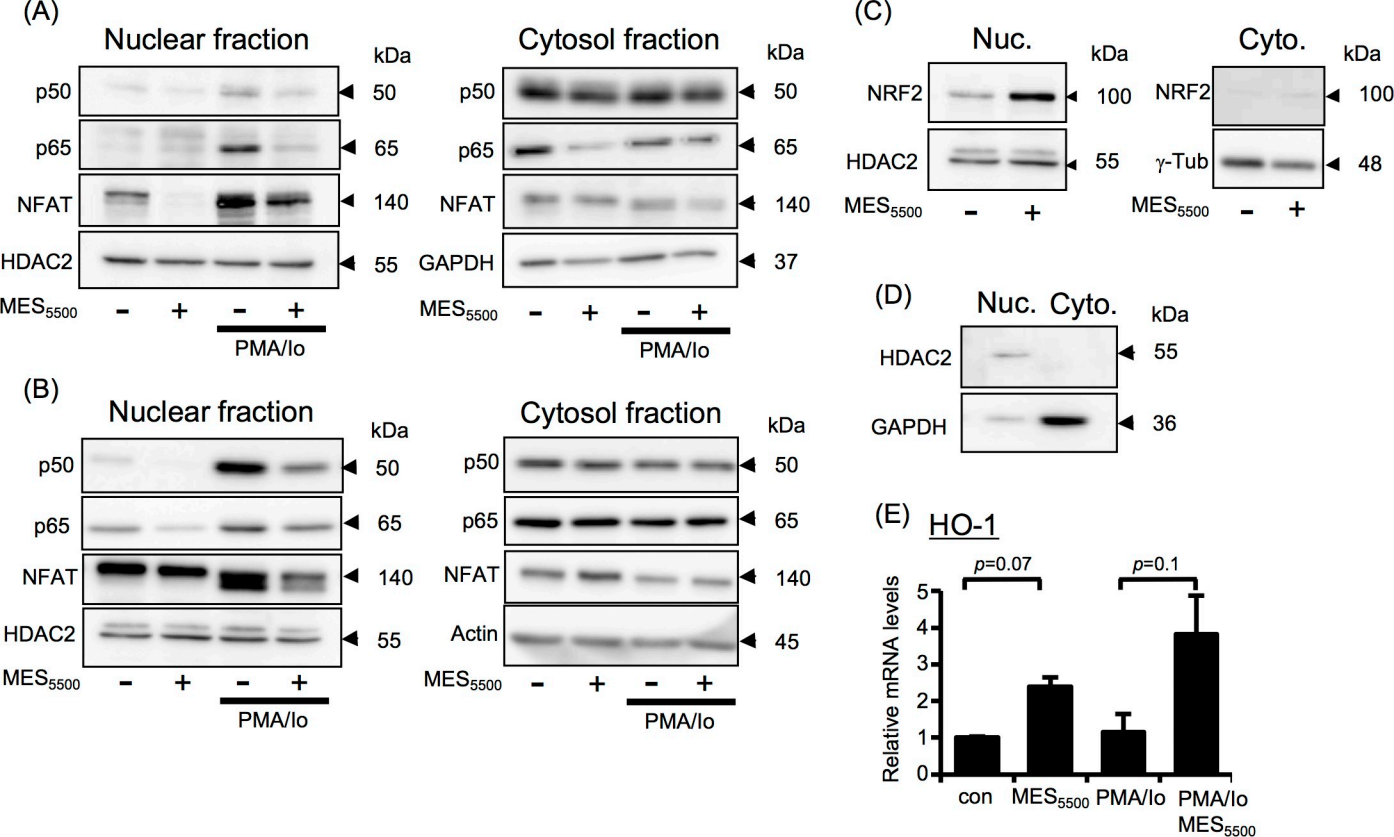
MES_5500_ shows anti-inflammatory effect *via* multiple pathways. Primary mouse splenocytes (A) and Jurkat T cells (B-E) were treated with MES_5500_. Medium was changed and cells were stimulated with PMA/Io. (A-D) Nuclear and cytosolic fractions were isolated and subjected to immunoblotting using the indicated antibodies. (E) Total RNA was extracted to detect HO-1 mRNA level. Data were normalized to the level of GAPDH mRNA (internal control). Data are presented as mean ± S.D. (n = 3 per group), assessed by Tukey-Kramer test (n.s.; not significant). The data shown are representative of 2 or more independent experiments.

To determine the mechanism underlying this effect, we focused on transcription factors that are known to control IL-2 and other cytokine gene expression in inflammation such as NF-κB, NFAT and NRF2 [21, 30, 31]. NF-κB and NFAT are mostly sequestered in the cytoplasm and, in the presence of an activator, are translocated to the nucleus to exert their transcriptional activating functions [21, 31]. Notably, MES_5500_ down-regulated the PMA/Io-induced nuclear expression of NF-κB p50 and p65 subunits and NFAT in nuclear lysates extracted from mouse primary splenocytes (Fig 4A, *left panel*) and Jurkat T cells (Fig 4B, *left panel*). No significant changes were observed in cytosolic fractions (Fig 4A and 4B, *right panels*). We also checked the effect of MES_5500_ on NRF2, which is a leucine zipper transcription factor that is essential in cellular responses. Studies showed that NRF2 activation inhibits NF-κB nuclear translocation and unveils anti-inflammatory response [30]. MES_5500_ increased the NRF2 protein expression in nuclear extracts isolated from Jurkat T cells treated with MES_5500_ for 10 min. (Fig 4C, *left panel*). NRF2 protein was not detected in cytosolic fraction (Fig 4C, *right panel*). To ensure that the nuclear fraction was not contaminated with cytosolic fraction, we checked the expression of proteins present only in nuclear fraction (HDAC2) or only in cytoplasm (GAPDH) (Fig 4D). MES_5500_ treatment tended to increase both the basal and PMA/Io-induced mRNA expression level of HO-1, a downstream mediator of NRF2 (Fig 4E).

Because in our study we showed that MES_5500_ can increase the mRNA level of IL-10, and IL-10 is known to modulate inflammation [32], we checked the protein level of IL-10 after MES_5500_ treatment. PathScan analysis showed an increase in protein levels of IL-10 (S4A Fig). To check whether MES_5500_ can exhibit its anti-inflammatory effects through IL10-PI3K signaling pathway [33], we investigated the effect of MES_5500_ on the activation of PI3K downstream factors mTOR and STAT3. MES_5500_ did not affect the phosphorylation of mTOR or STAT3 (S4B Fig), indicating that its anti-inflammatory effect is independent of the PI3K signaling pathway. Overall, our data indicated that the anti-inflammatory effect of MES_5500_ involves inhibiting the nuclear translocation of NF-κB and NFAT, and activating the NRF2/HO-1 axis.

### The effect of MES_5500_ on cell-free culture media

**Fig 5:**
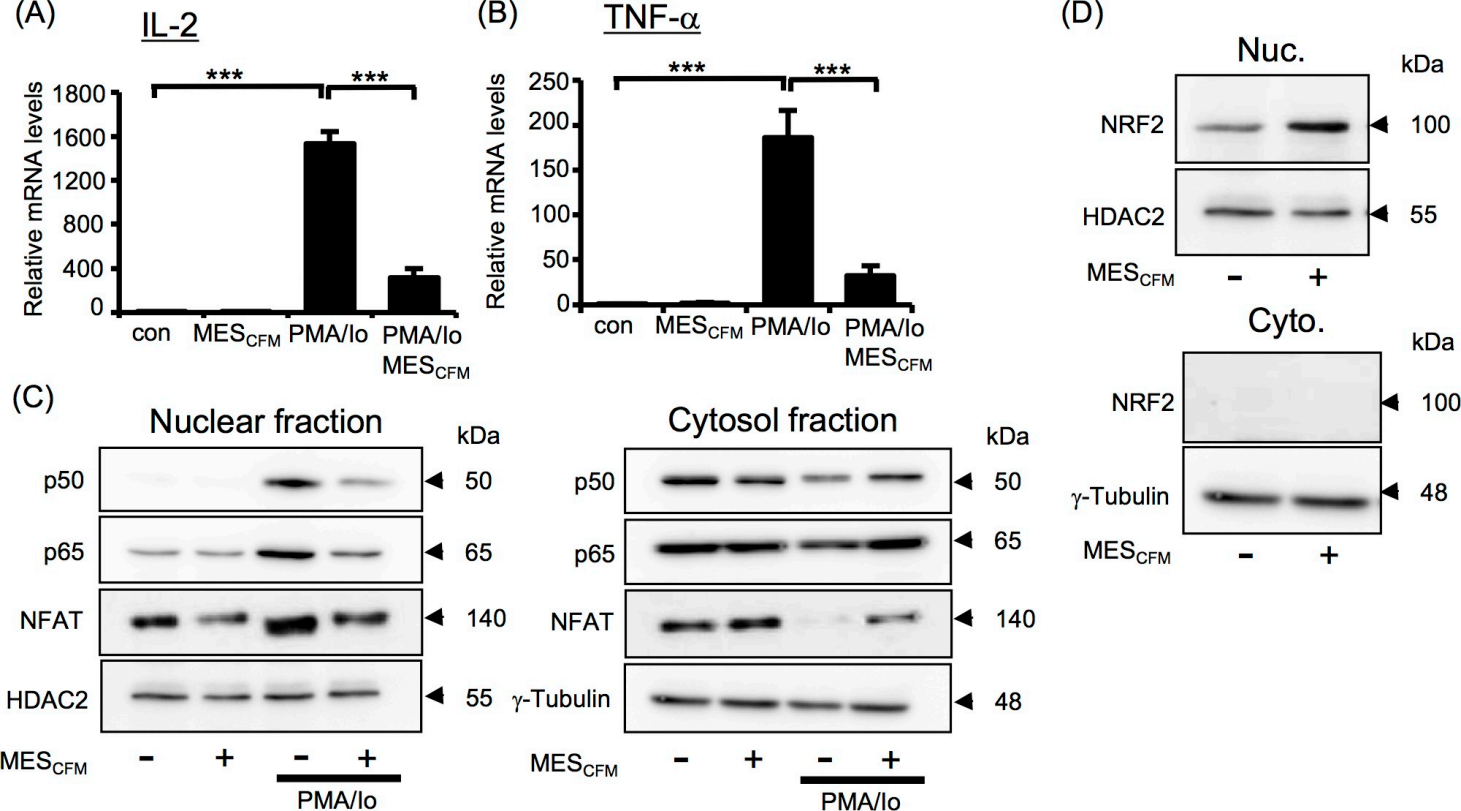
MES_5500_-treated cell-free culture media (MES_CFM_) shows immunosuppressive effect. (A, B) IL-2 and TNF-α mRNA levels were analyzed in Jurkat T cells treated with MES_CFM_ and stimulated with PMA/Io. Data were normalized to the level of GAPDH mRNA (internal control). Data are presented as mean ± S.D. (n = 3 per group) ***P<0.001 assessed by Tukey-Kramer test. (C-D) Immunoblotting of nuclear and cytosolic fractions from Jurkat T cells treated with MES_CFM_ and/or PMA/Io. The data shown are representative of 2 or more independent experiments.

To check whether the effect of MES_5500_ is directly on the cells or *via* production of some compounds in culture media or serum (*in vivo* system), we treated cell-free culture medium, with MES_5500_ for 10 min, termed as MES_CFM_, and added it to Jurkat T cells for 10 min at 37°C. Medium was changed, and cells were stimulated with PMA/Io for 3 hr. Interestingly, MES_CFM_, similar to MES_5500_, directly showed immunosuppressive effect by inhibiting PMA/Io-induced overproduction of IL-2 (Fig 5A) and TNF-α (Fig 5B). Western blotting analysis revealed that MES_CFM_ not only suppressed nuclear translocation of NF-κB and NFAT (Fig 5C, *left panel*), but also contributed to their exit from nucleus to cytoplasm (Fig 5C, *right panel*). Consistently, MES_CFM_ increased NRF2 expression in nuclear fraction, while it was not detected in cytosolic fraction (Fig 5D). These results suggested the possible existence of some compounds produced by MES_5500_ that are responsible for its anti-inflammatory effect.

### MES_5500_ induces the production of H_2_O_2_ *in vitro* and slight increase in serum H_2_O_2_ *in vivo*

**Fig 6:**
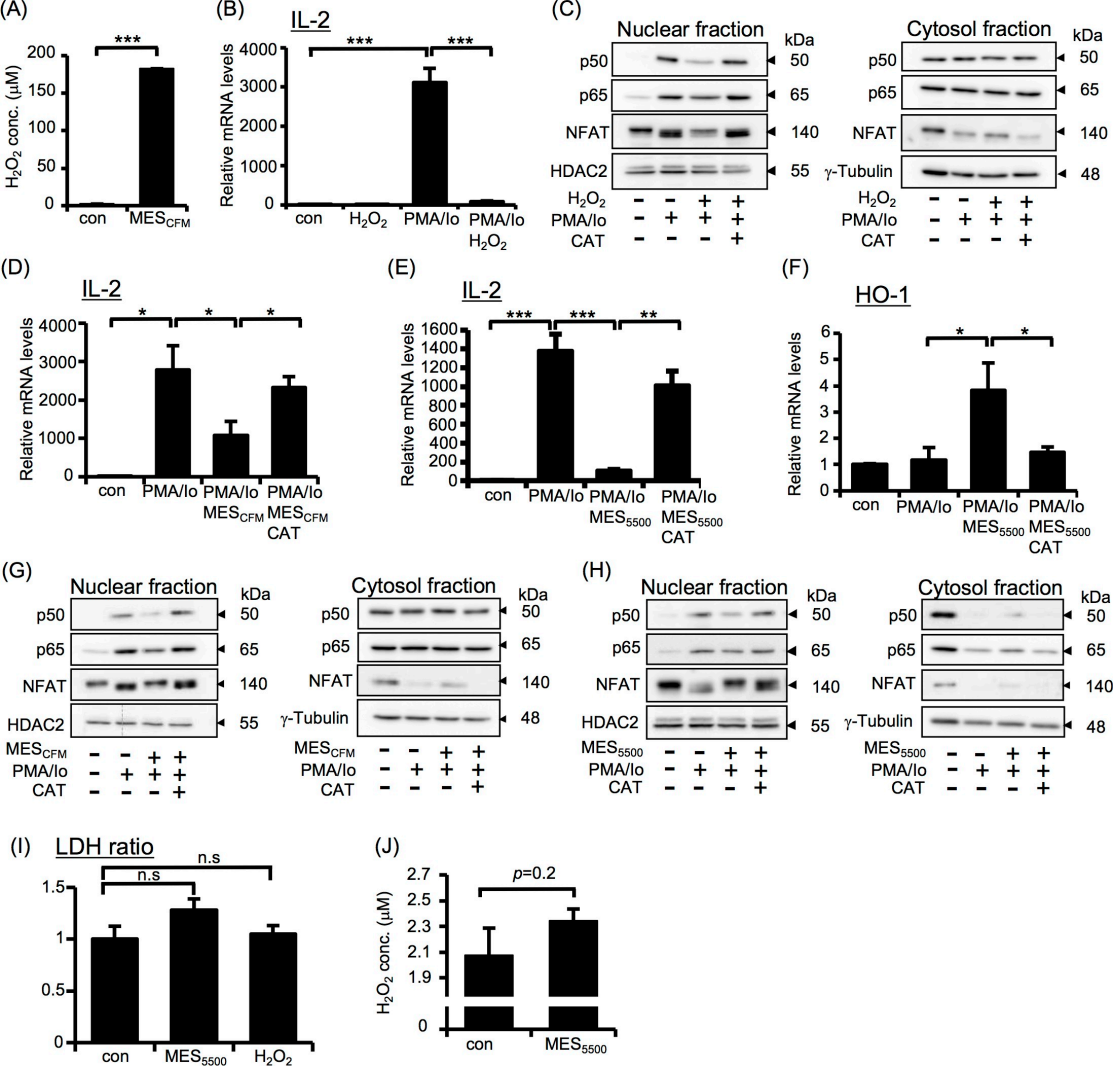
MES_5500_ induces production of H_2_O_2_, which is partly responsible for its anti-inflammatory effect. (A) H_2_O_2_ concentration in MES_CFM_. (B) IL-2 mRNA level in Jurkat T cells treated with H_2_O_2_ and stimulated with PMA/Io. (C) Immunoblots of nuclear and cytosolic fractions of Jurkat cells incubated with catalase-treated H_2_O_2_ and stimulated with PMA/Io. (D-F) Jurkat T cells were treated with MES_CFM_ (D) or MES_5500_ (E, F) with or without catalase (CAT), and stimulated with PMA/Io. Total RNA was analyzed for the indicated genes. (G, H) Immunoblots of nuclear and cytosolic fractions of cells treated with MES_CFM_ (G) or MES_5500_ (H) with or without catalase, and stimulated with PMA/Io. (I) Jurkat T cells were treated with MES_5500_ or H_2_O_2_ for 10 min and medium was changed. After 24 hr, LDH leakage from cells was measured. (B, D-F) Data were normalized to the level of GAPDH mRNA (internal control). Data are presented as mean ± S.D. (n = 3 per group). (A, F, I) *P<0.05, ***P<0.001 assessed by Wilcoxon test. (B, D, E) *P<0.05, **P<0.01, ***P<0.001 assessed by Tukey-Kramer test. (n.s.; not significant). (J) BALB/c mice (13 weeks old) were treated with MES_5500_ for 30 min, and serum was obtained to measure H_2_O_2_ concentration. Data are presented as mean ± S.D. (n = 4 for control group and n = 5 for MES_5500_-treated group) assessed by Wilcoxon test. The data shown are representative of 2 or more independent experiments.

Our investigations revealed that the compound responsible for the anti-inflammatory effect of MES_5500_ is neither protein nor nucleic acid in origin, and is independent of cell type, media and pH (S5 Fig). Because it was previously reported that electrical stimulation produces H_2_O_2_ [34], we determined the involvement of H_2_O_2_ in the effect of MES_5500_. We quantified H_2_O_2_ in MES_CFM_ using H_2_O_2_ assay kit. H_2_O_2_ was detected at a concentration of approximately 200 μM in MES_CFM_ (Figs 6A and S5E). To check whether the H_2_O_2_ production is linked to the immunosuppressive effect of MES_5500_, we treated Jurkat T cells with 200 μM H_2_O_2_ for 10 min, changed the medium and stimulated the cells with PMA/Io. Similar to the effect of MES_5500_, 200 μM H_2_O_2_ suppressed the overproduction of IL-2 after PMA/Io treatment (Fig 6B). H_2_O_2_ also inhibited the PMA/Io-induced nuclear translocation of NF-κB and NFAT (Fig 6C, *left panel*) and kept the NFAT in cytosolic fraction (Fig 6C, *right panel*). The effect of H_2_O_2_ on the localizations of NF-κB and NFAT was abrogated by catalase. Next, we further checked the effect of catalase, an enzyme that catalyzes the decomposition of H_2_O_2_ to water and oxygen, on the immunosuppressive effects of MES_CFM_ and MES_5500_. Jurkat T cells were untreated or treated with MES_CFM_ or MES_5500_ for 10 min with or without catalase. Medium was changed, and cells were then stimulated with PMA/Io. Catalase treatment abrogated the effect of both MES_CFM_ and MES_5500_ on PMA/Io-induced IL-2 expression (Fig 6D and 6E). Catalase also inhibited the increase in HO-1 mRNA level after MES_5500_ treatment (Fig 6F). Catalase abrogated the effects of MES_CFM_ and MES_5500_ on the inhibition of nuclear translocation of NF-κB and NFAT (Fig 6G and 6H, *left panels*) and prevented the relocation of NF-κB and NFAT into cytosolic fraction (Fig 6G and 6H, *right panels*), indicating the involvement of H_2_O_2_ in the effects of MES_5500_. To check possible cytotoxic effect of MES_5500_, we performed lactate dehydrogenase (LDH) assay. Cells were treated with MES_5500_ or H_2_O_2_ for 10 min, medium was changed, and cells were incubated at 37°C for 24 hr. There was no significant change between control, MES_5500_-treated and H_2_O_2_-treated groups (Fig 6I), indicating the absence of toxic effect from MES_5500_ treatment.

To determine whether MES_5500_ also induces production of H_2_O_2_ *in vivo*, we measured the H_2_O_2_ concentration in blood serum of BALB/c mice treated with MES_5500_ for 30 min. Although not statistically significant, we detected a slight increase in serum H_2_O_2_ after MES_5500_ treatment (Fig 6J). Furthermore, to confirm the H_2_O_2_ involvement in *in vivo* system, we checked NRF2 level in livers of MES_5500_-treated ConA model. MES_5500_ treatment significantly increased the level of NRF2 protein in mouse liver (S6A and S6B Fig). MES_5500_ treatment also increased the level of H_2_O_2_ in the serum of mice intraperitoneally injected with catalase inhibitor (ATZ) (S6C Fig) but because of the short half-life of H_2_O_2_ in the blood, it is challenging to get any significant change. However, previous studies suggest that a moderate increase of H_2_O_2_ in the physiological range acts as a signaling molecule, and is sufficient to regulate molecular pathways [35, 36]. Therefore, we can postulate that this slight increase in serum H_2_O_2_ may be biologically relevant.

## Discussion

**Fig 7:**
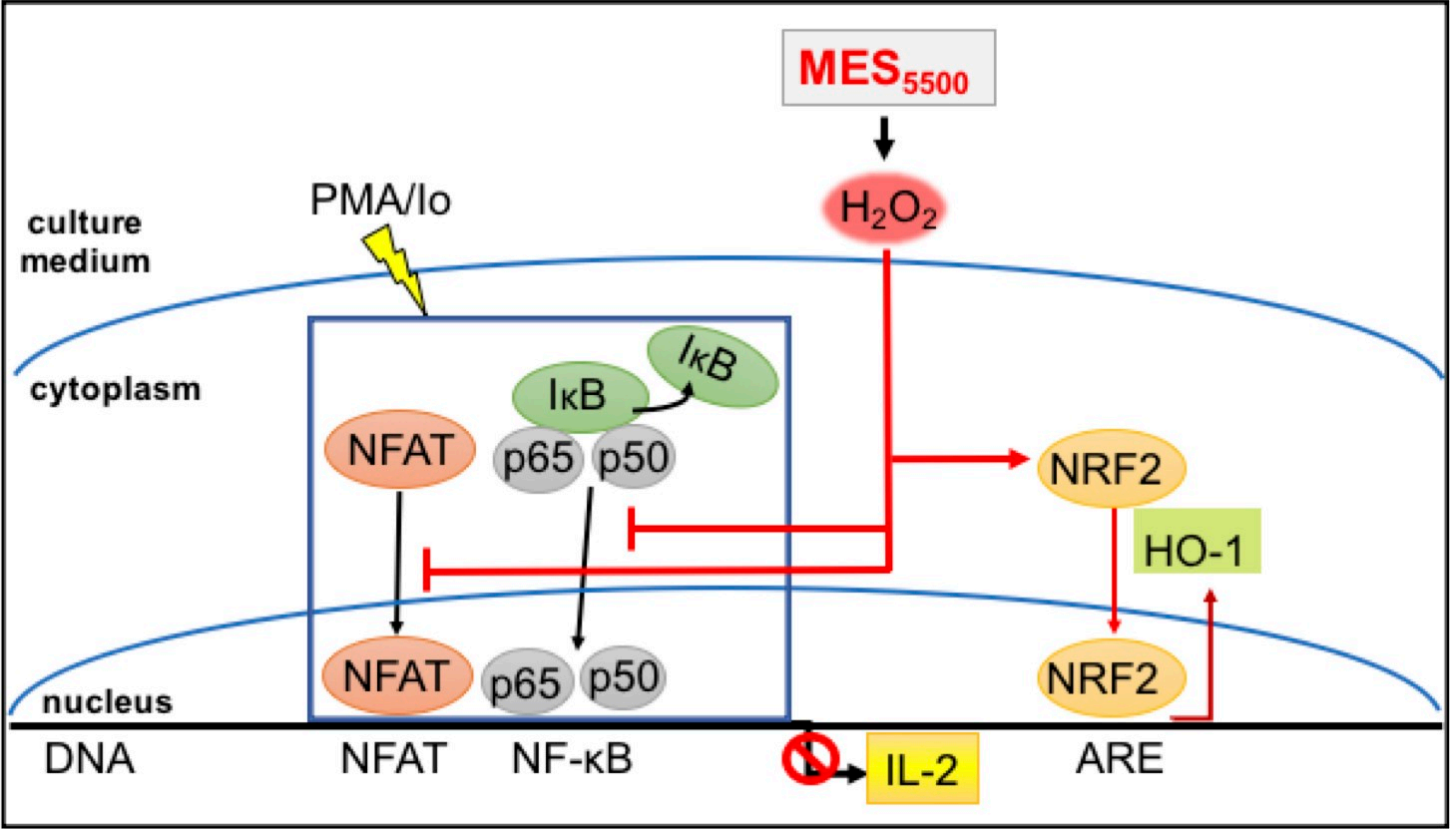
The schematic diagram of MES_5500_ mechanism. Therapeutic use of electric current has been practiced for various kinds of inflammation, and the mechanism was thought to be its analgesic effect or by blood flow improvement [38]. It is well known that in an electrolytic reaction, electrical current generates H_2_O_2_ from water [39], and as shown in our study, this reaction might also occur in electrical stimulation used for therapeutic purposes. While many reports indicate that reactive oxygen species (ROS), such as H_2_O_2_, exerts pro-inflammatory effects through activation of NF-κB, more recent studies have shown that ROS are not only responsible for inducing inflammation, but also have potent anti-inflammatory properties [40, 41]. Increase in intracellular concentration of H_2_O_2_ have been demonstrated to diminish TLR4-induced activation of NF-κB and production of pro-inflammatory cytokines in neutrophils, epithelial cells and other cell populations [42]. Aside from T cells and primary splenocytes, MES_5500_ was also able to reduce the inflammatory cytokine expression in other cells such as MG6 (microglia), SHSY5Y (neuroblastoma lineage) (S5 Fig) and HeLa cells (S7 Fig). MES_5500_ did not have any effect at the basal state of these cells, indicating the safety of the treatment, and it modulated the excess reaction after inflammatory reagent stimulation. Moreover, MES_5500_ reduced the induction of IL-1β upon stimulation of these cells with lipopolysaccharide (LPS), an immune activator that binds to Toll-like receptor (TLR) 4 (S5A–S5C Fig). These data consolidate our findings that MES_5500_ has immune suppressor effect.

In our continued effort to find optimal conditions of MES towards its application for refractory disease conditions [8], we investigated here the impact of high frequency pulse currents on inflammation. Because our previous works mostly focused on low frequency current (MES_55_) [7, 9, 37] (among others), the present study revealed for the first time the usefulness of high frequency pulse current (MES_5500_) specifically towards modulating cytokine overexpression in the context of PMA/Io- and ConA-stimulated immune response. The excessive production of cytokines can lead to inflammatory diseases. Thus, suppressing excessive production of inflammatory cytokines is effective in the treatment of various inflammatory-related diseases. The main mechanism by which MES_5500_ inhibited the induction of inflammatory response is the suppression of PMA/Io-induced nuclear translocation of NFAT and NF-κB and the activation of NRF2/HO-1 axis with the involvement of H_2_O_2_ that is produced by MES_5500_ (Fig 7).

We tried to analyze the activation pattern of splenocytes from the mice treated with MES_5500_ stimulation, we performed *in vivo* MES_5500_ treatment and *ex vivo* stimulation of splenocytes with PMA/Io (S8A Fig). We observed that the *ex vivo* PMA/Io-induced increase of IL-2 and IL-8 mRNA expression levels were not suppressed by the *in vivo* treatment with MES_5500_ (S8B and S8C Fig). IL-10 mRNA level was increased in MES_5500_-treated mice although the change was not statistically significant (S8D Fig). The expression of TGF-β was significantly reduced by *ex vivo* PMA/Io treatment, which was only slightly elevated in splenocytes of MES_5500_-treated mice (S8E Fig). These data indicated that activation of splenocytes by MES_5500_ *in vivo* may be transient, and is not sufficient to induce the suppression of inflammatory cytokines that were strongly activated by *ex vivo* treatment with PMA/Io. But because MES_5500_ treatment was able to suppress *in vivo* inflammation (Fig 2A–2L), which is a more realistic physiological scenario, then MES5500 has effective anti-inflammation property. The fact that splenocytes are only activated temporarily *in vivo* supports well the general non-toxicity of MES_5500_.

Compared to the direct treatment with MES_5500_ to cells, MES_CFM_ treatment shows milder immunosuppressive effect. Moreover, catalase treatment abrogated the effect of MES_CFM_ and recovered PMA/Io-induced production of IL-2 to 90%, indicating that H_2_O_2_ mostly mediated the immunomodulatory effect of MES_CFM_. In contrast, the effect of catalase on direct MES_5500_ treatment recovered PMA/Io-induced IL-2 production only up to 70% (Fig 6E). This result indicated that the effects of direct treatment with MES_5500_ to the cells is partly independent of H_2_O_2_. This may not be surprising considering that pulsed electrical stimulation affects several cellular signaling pathways [5], however, the mechanistic details in our study need further investigation.

Regarding the *in vivo* results, although we showed that MES_5500_ slightly increased H_2_O_2_ levels in blood serum of mice (Figs 6J and S6C), more explorations are needed to precisely know the distribution of H_2_O_2_ *in vivo*. Such low level of H_2_O_2_ can be explained by the abundance of catalase in tissues and blood that causes the short half-life of H_2_O_2_ [43]. The complicated *in vivo* system and the ability of MES_5500_ to recruit multiple signaling pathways make it challenging to identify the precise mechanism *in vivo*. In this study, we only focused on ConA-induced immune related liver damage. Our further goal is to check the therapeutic effect of MES_5500_ on other well-established T cell-specific immune disease models, and to fully understand the T cell immunosuppressive effect of MES_5500_. Unlike other studies in which electrical stimulation induces production of high concentration H_2_O_2_ to kill bacteria [44], or induces apoptosis in cancer cells [45], we used mild electrical stimulation to generate moderate levels of H_2_O_2_ that do not lead to cell damage. The function generator used in our study that delivers MES_5500_ can also be used for animal studies. In conclusion, the immunosuppression triggered by MES_5500_ may be effective for the treatment of inflammation-related diseases promoted by excess inflammatory cytokine production. MES_5500_ may also be considered as adjunctive therapy for existing immunosuppressive drugs for various autoimmune diseases.

## Supporting information

S1 Fig MES_5500_ shows characteristic anti-inflammatory effects. (PDF)

S2 Fig MES_5500_ reduces spleen size and inflammation in ConA-treated mice. (PDF)

S3 Fig MES_5500_ suppresses ConA-stimulated overexpression of inflammatory cytokines in Jurkat T cells and primary splenocytes. (PDF)

S4 Fig MES_5500_ increases protein levels of IL-10, but does not have effect on PI-3K downstream molecules. (PDF)

S5 Fig Compound responsible for anti-inflammatory effect of MES_5500_ is independent on cell type, culture media. (PDF)

S6 Fig MES_5500_ induces production of H_2_O_2_ *in vivo*. (PDF)

S7 Fig MES_5500_ shows anti-inflammatory effect on HeLa cells. (PDF)

S8 Fig MES_5500_ treatment in mice does not ameliorate *ex vivo* PMA/Io-induced overexpression of inflammatory cytokines in primary splenocytes. (PDF)

S1 Table Antibodies used for Western blotting. (PDF)

S1 Raw images (ZIP)
