# Supplementary material for: A novel condition of mild electrical stimulation exerts immunosuppression via hydrogen peroxide production that controls multiple signaling pathway
Source: PLoS One. 2020 Jun 22;15(6):e0234867. doi: 10.1371/journal.pone.0234867 (PMC7307747; doi:10.1371/journal.pone.0234867)
Supplement: S1 Fig — (PDF) [file pone.0234867.s001.pdf]

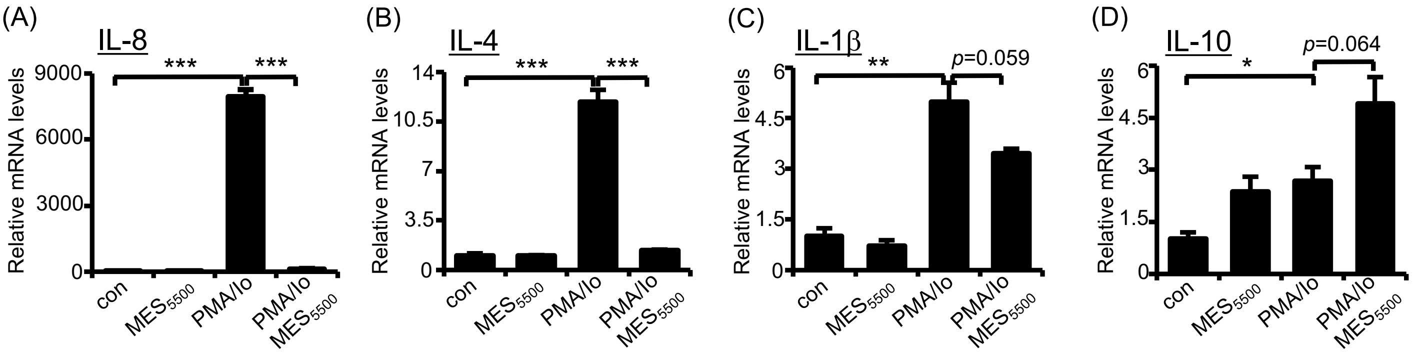

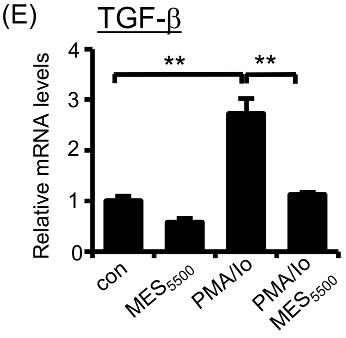

S1 Fig. MES_{5500} shows characteristic anti-inflammatory effects.
(A-E) Jurkat cells were treated with MES_{5500} at 2V/cm for 10 min. After treatment, the medium was changed and cells were stimulated with PMA/Io for 3 hr at 37 ^{o}C. Total RNA was extracted and subjected to quantitative RT-PCR to detect the indicated genes. (A-E) Data were normalized to the level of 18s mRNA (internal control). Data are presented as mean ± S.D. (n=3 per group). *$P < 0.05$, **$P<0.01$, ***$P<0.001$ assessed by Tukey-Kramer test. The data shown are representative of 2 or more independent experiments.
