# Supplementary material for: A novel condition of mild electrical stimulation exerts immunosuppression via hydrogen peroxide production that controls multiple signaling pathway
Source: PLoS One. 2020 Jun 22;15(6):e0234867. doi: 10.1371/journal.pone.0234867 (PMC7307747; doi:10.1371/journal.pone.0234867)
Supplement: S2 Fig — (PDF) [file pone.0234867.s002.pdf]

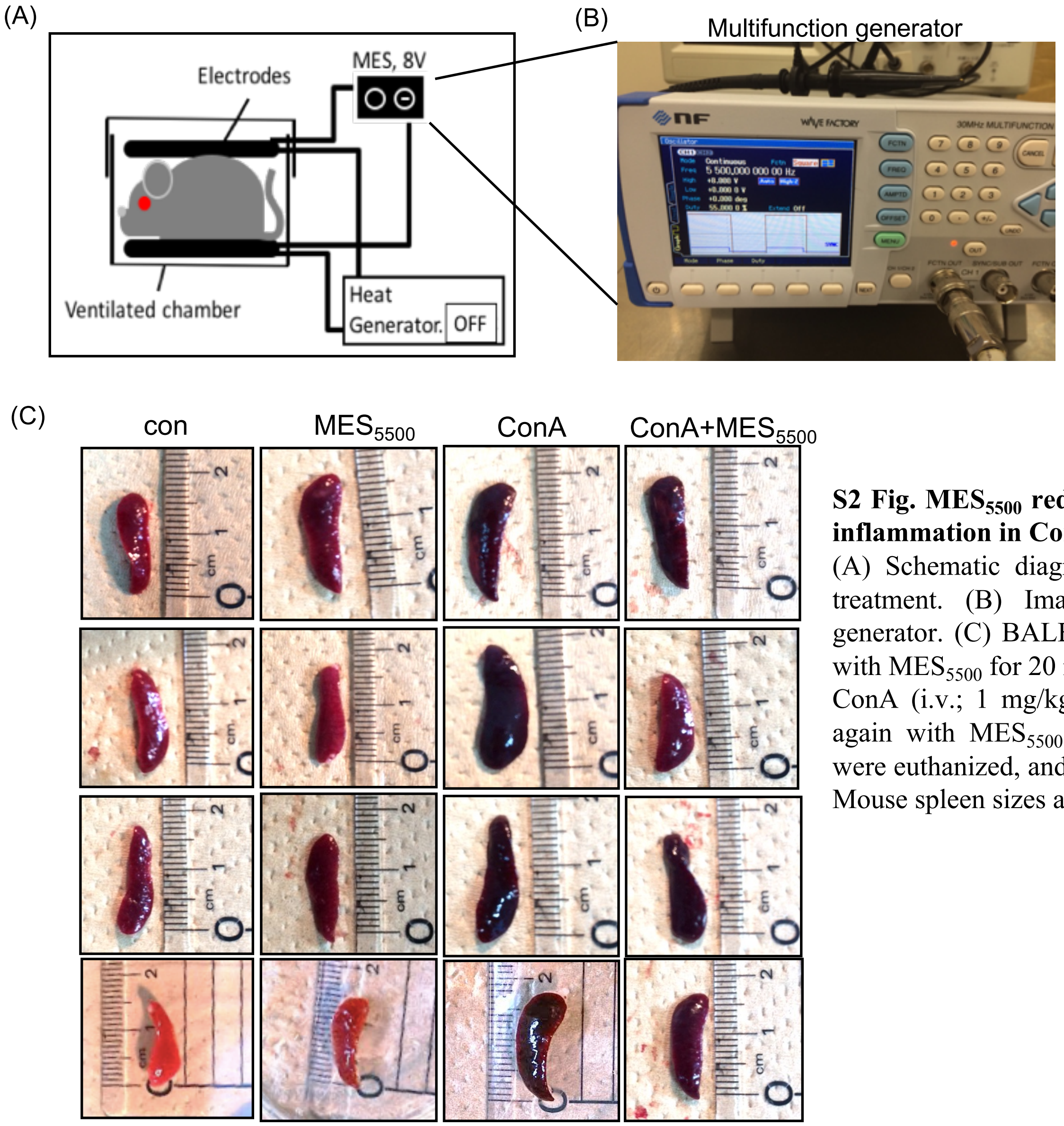

S2 Fig. MES_{5500} reduces spleen size and inflammation in ConA-treated mice.
(A) Schematic diagram of *in vivo* MES treatment. (B) Image of multifunction generator. (C) BALB/c mice were treated with MES_{5500} for 20 min, and injected with ConA (i.v.; 1 mg/kg). Mice were treated again with MES_{5500}. Four hr later, mice were euthanized, and spleen was collected. Mouse spleen sizes are shown.
