# Supplementary material for: A novel condition of mild electrical stimulation exerts immunosuppression via hydrogen peroxide production that controls multiple signaling pathway
Source: PLoS One. 2020 Jun 22;15(6):e0234867. doi: 10.1371/journal.pone.0234867 (PMC7307747; doi:10.1371/journal.pone.0234867)
Supplement: S4 Fig — (PDF) [file pone.0234867.s004.pdf]

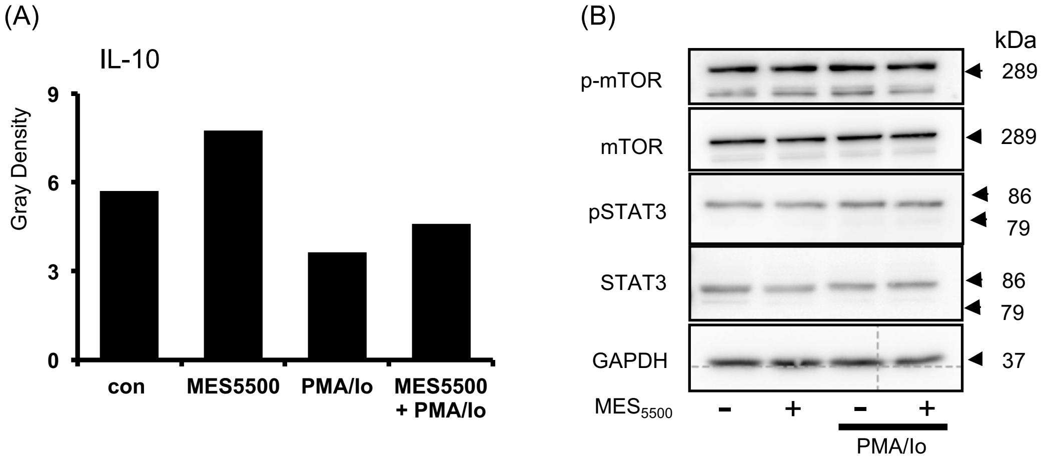

S4 Fig. MES_{5500} increases protein level of IL-10, but does not have effect on PI3K downstream molecules. (A-B) Jurkat T cells were treated with MES_{5500} at 2V/cm for 10 min. (A) After treatment, the medium was changed and cells were stimulated with PMA/Io for 6 hr at 37 ^{o}C, and PathScan analysis was performed according to manufacturer's protocol (*Cell Signaling Technology*). (B) Western blotting was performed on whole cells lysates from cells stimulated with PMA/Io for 3 hr at 37 ^{o}C.
