# Supplementary material for: A novel condition of mild electrical stimulation exerts immunosuppression via hydrogen peroxide production that controls multiple signaling pathway
Source: PLoS One. 2020 Jun 22;15(6):e0234867. doi: 10.1371/journal.pone.0234867 (PMC7307747; doi:10.1371/journal.pone.0234867)
Supplement: S5 Fig — (PDF) [file pone.0234867.s005.pdf]

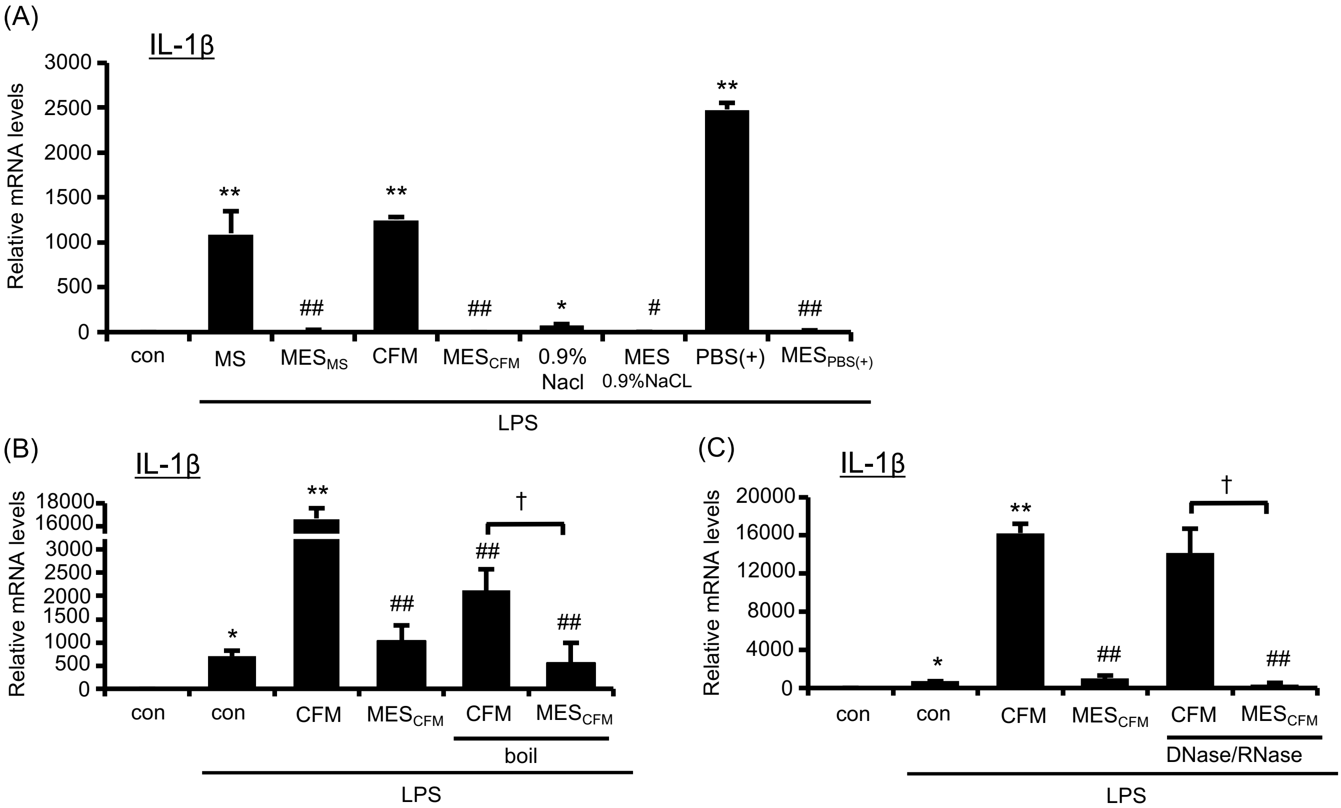

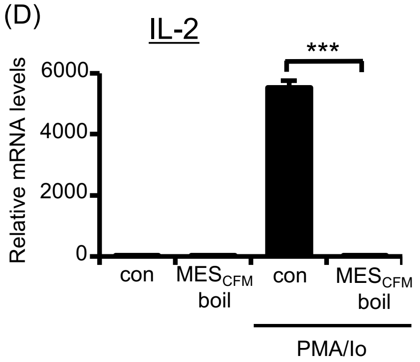

(E)

Electrical Current	H_2O_2 conc. (μm)
AC	7
55 pps	0
550 pps	2.3
5500 pps	180

S5 Fig. Compound responsible for anti-inflammatory effect of MES_{5500} is independent on cell type, culture media.

(A) MG6 cells with medium (MS), DMEM high glucose (CFM), 0.9 % NaCl and PBS(+) were treated with MES_{5500} at 2 V/cm for 30 min. Then media were transferred to MG6 cells, which were cultured in serum-free media for 4 hr at 37 ^{o}C, and cells were stimulated with 100 ng/ml LPS for 4 hr at 37 ^{o}C. Total RNA was extracted and analyzed to detect IL-1β mRNA. (B, C) SHSY5Y cells were treated with MES_{5500} at 2 V/cm for 30 min and were incubated for 4 hr at 37 ^{o}C without medium change. The supernatants from MES_{5500} treated or non-treated cells were (B) boiled for 15 min at 100 ^{o}C, (C) treated with 2 mg/ml Dnase/Rnase for 30 min at 37 ^{o}C, and added to MG6 cells (2nd cells), which were cultured in serum-free media for 4 hr, and cells were stimulated with 100 ng/ml LPS for 4 hr. Total RNA of 2nd cells was extracted and analyzed to detect IL-1β. Data were normalized to the level of GAPDH (internal control). (D) Cell-free culture media was treated with MES_{5500} for 10 min (MES_{CFM}), and boiled. After cooling, MES_{CFM} was added to Jurkat T cells for 10 min. The medium was changed and cells were stimulated with PMA/Io for 3 hr. Total RNA was extracted and analyzed to detect IL-2 mRNA levels. Data were normalized to the level of GAPDH (internal control). Data are presented as mean ± S.D. (n=3 per group). *P<0.05, **P<0.01, non-treated vs. LPS-treated; #P<0.05, ##P<0.01, LPS-treated vs. MES_{5500}; †P<0.05, B and C as indicated. Data were assessed by ANOVA with Tukey-Kramer test. (E) H_2O_2 concentration in cell media after 10 min of electrical stimulation with different currents. AC, alternating current.
