# Supplementary material for: A novel condition of mild electrical stimulation exerts immunosuppression via hydrogen peroxide production that controls multiple signaling pathway
Source: PLoS One. 2020 Jun 22;15(6):e0234867. doi: 10.1371/journal.pone.0234867 (PMC7307747; doi:10.1371/journal.pone.0234867)
Supplement: S6 Fig — (PDF) [file pone.0234867.s006.pdf]

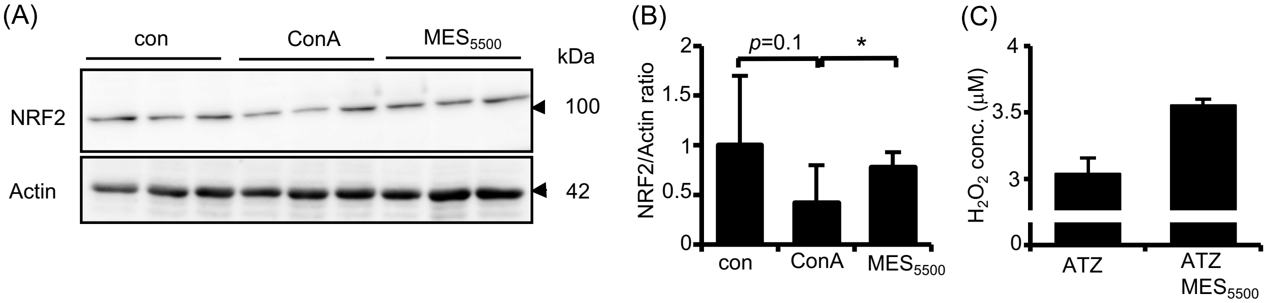

S6 Fig. MES_{5500} induces production of H_2O_2 *in vivo*.

(A) Immunoblotting of liver lysates isolated from BALB/c mice treated with MES_{5500} and ConA. (B) Quantification of blots in (A). (C) BALB/c mice (6 weeks old) were injected with 3-amino-1,2,4-triazole (ATZ; 1g/kg; i.p.). After 24 h, mice were treated with MES_{5500}. Blood serum was obtained to measure H_2O_2 concentration. Data are presented as mean ± S.D. (n=4 for control group and n=4 for MES_{5500}-treated group) assessed by Wilcoxon test. The data shown are representative of 2 or more independent experiments.
