# Supplementary material for: A novel condition of mild electrical stimulation exerts immunosuppression via hydrogen peroxide production that controls multiple signaling pathway
Source: PLoS One. 2020 Jun 22;15(6):e0234867. doi: 10.1371/journal.pone.0234867 (PMC7307747; doi:10.1371/journal.pone.0234867)
Supplement: S7 Fig — (PDF) [file pone.0234867.s007.pdf]

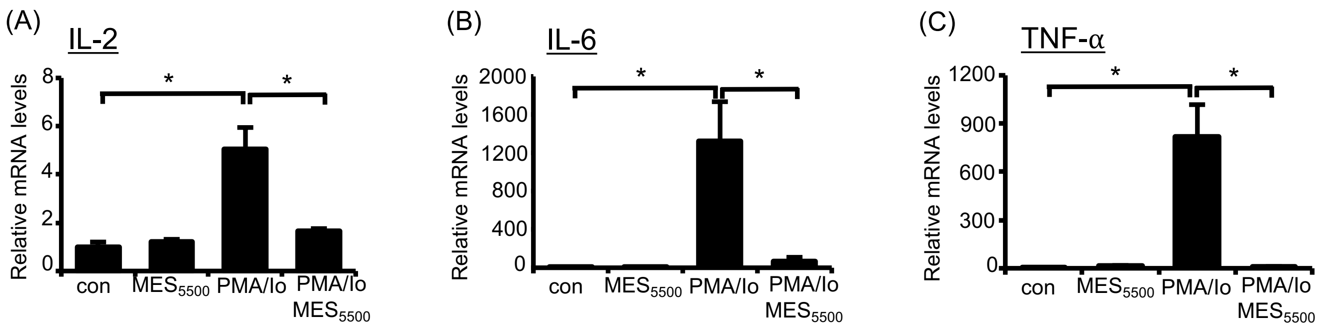

S7 Fig. MES_{5500} shows anti-inflammatory effect on HeLa cells.

(A-C) HeLa cells were treated with MES_{5500}, and stimulated with PMA/Io. Total RNA was extracted and analyzed to detect the indicated genes. Data were normalized to the level of 18s mRNA (internal control). Data are presented as mean ± S.D. (n=3 per group). *$P < 0.05$ assessed by Tukey-Kramer test. The data shown are representative of 2 or more independent experiments.
