# Supplementary material for: A novel condition of mild electrical stimulation exerts immunosuppression via hydrogen peroxide production that controls multiple signaling pathway
Source: PLoS One. 2020 Jun 22;15(6):e0234867. doi: 10.1371/journal.pone.0234867 (PMC7307747; doi:10.1371/journal.pone.0234867)
Supplement: S8 Fig — (PDF) [file pone.0234867.s008.pdf]

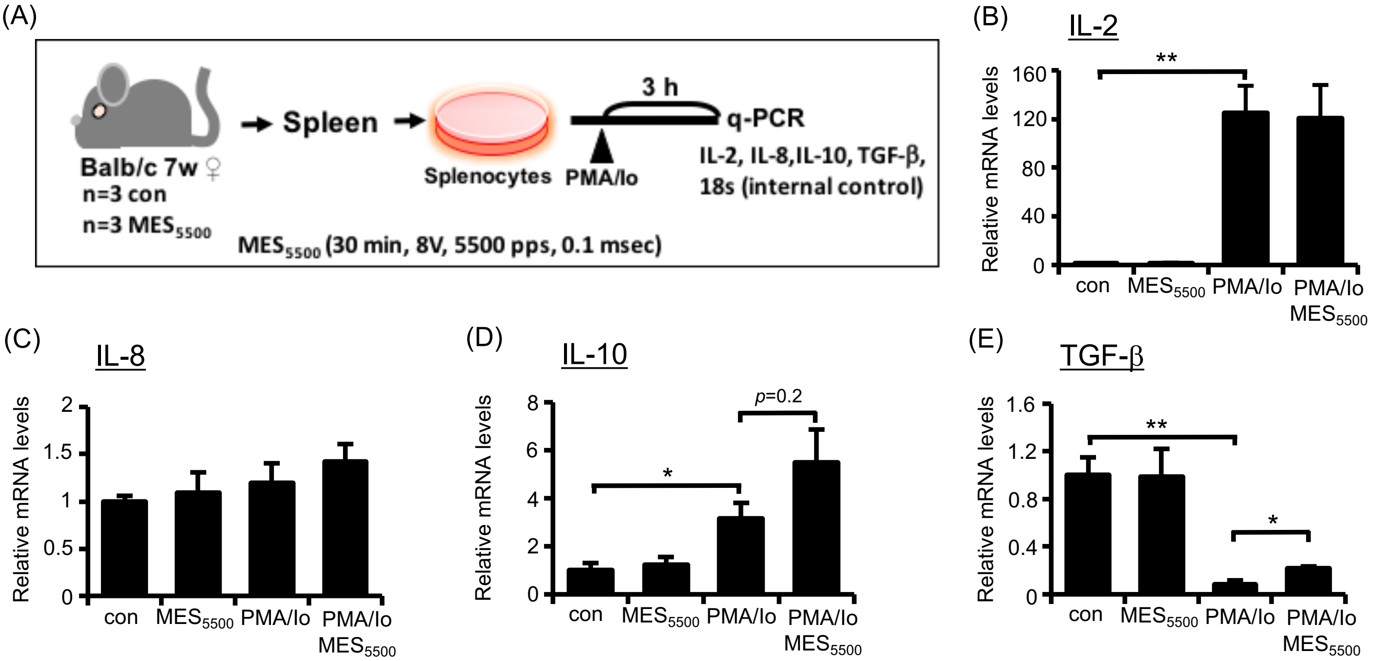

S8 Fig. MES_{5500} treatment in mice does not ameliorate *ex vivo* PMA/Io-induced overexpression of inflammatory cytokines in primary splenocytes.

(A-E) BALB/c mice were treated with MES_{5500} for 30 min. Then mice were euthanized and spleen was collected for splenocyte isolation. Splenocytes were treated with PMA/Io for 3 hr at 37 ^{o}C. (A) Schematic diagram of the experimental plan. (B-E) Total RNA was extracted and subjected to quantitative RT-PCR to detect the indicated genes. Data were normalized to the level of 18s mRNA (internal control). Data are presented as mean ± S.D. (n=3 per group). $*P < 0.05$, $**P<0.01$ assessed by Tukey-Kramer test. The data shown are representative of 2 or more independent experiments.
