# Supplementary material for: A novel condition of mild electrical stimulation exerts immunosuppression via hydrogen peroxide production that controls multiple signaling pathway
Source: PLoS One. 2020 Jun 22;15(6):e0234867. doi: 10.1371/journal.pone.0234867 (PMC7307747; doi:10.1371/journal.pone.0234867)
Supplement: S1 Table — (PDF) [file pone.0234867.s009.pdf]

S1 Table. Antibodies used for Western blotting

1st antibody	Vendor (Company)	Dilution	2nd antibody	Vendor (Company)	Dilution
p50	Santa Cruz Biotechnology	1:1000	Mouse monoclonal IgG_1	Cell Signaling Technology	1:5000
p65	Santa Cruz Biotechnology	1:1000	Mouse monoclonal IgG_1	Cell Signaling Technology	1:5000
NFAT	Cell Signaling Technology	1:1000	Rabbit Ab	Cell Signaling Technology	1:5000
γ-Tubulin	Santa Cruz Biotechnology	1:1000	Mouse monoclonal IgG_1	Cell Signaling Technology	1:5000
Actin	Santa Cruz Biotechnology	1:1000	Goat polyclonal IgG	Santa Cruz Biotechnology	1:5000
NRF2	Abcam	1:500	Rabbit polyclonal IgG	Cell Signaling Technology	1:1000
HDAC2	Santa Cruz Biotechnology	1:1000	Rabbit polyclonal IgG	Cell Signaling Technology	1:5000
mTOR	Cell Signaling Technology	1:1000	Rabbit mAb	Cell Signaling Technology	1:5000
p-mTOR	Cell Signaling Technology	1:1000	Rabbit Ab	Cell Signaling Technology	1:5000
STAT3	Santa Cruz Biotechnology	1:1000	Rabbit polyclonal IgG	Cell Signaling Technology	1:5000
p-STAT3	Santa Cruz Biotechnology	1:1000	Rabbit polyclonal IgG	Cell Signaling Technology	1:5000
GAPDH	Santa Cruz Biotechnology	1:1000	Mouse monoclonal IgG_1	Cell Signaling Technology	1:5000
