# Supplementary figures and images for: A novel condition of mild electrical stimulation exerts immunosuppression via hydrogen peroxide production that controls multiple signaling pathway
Source: PLoS One. 2020 Jun 22;15(6):e0234867. doi: 10.1371/journal.pone.0234867 (PMC7307747; doi:10.1371/journal.pone.0234867)

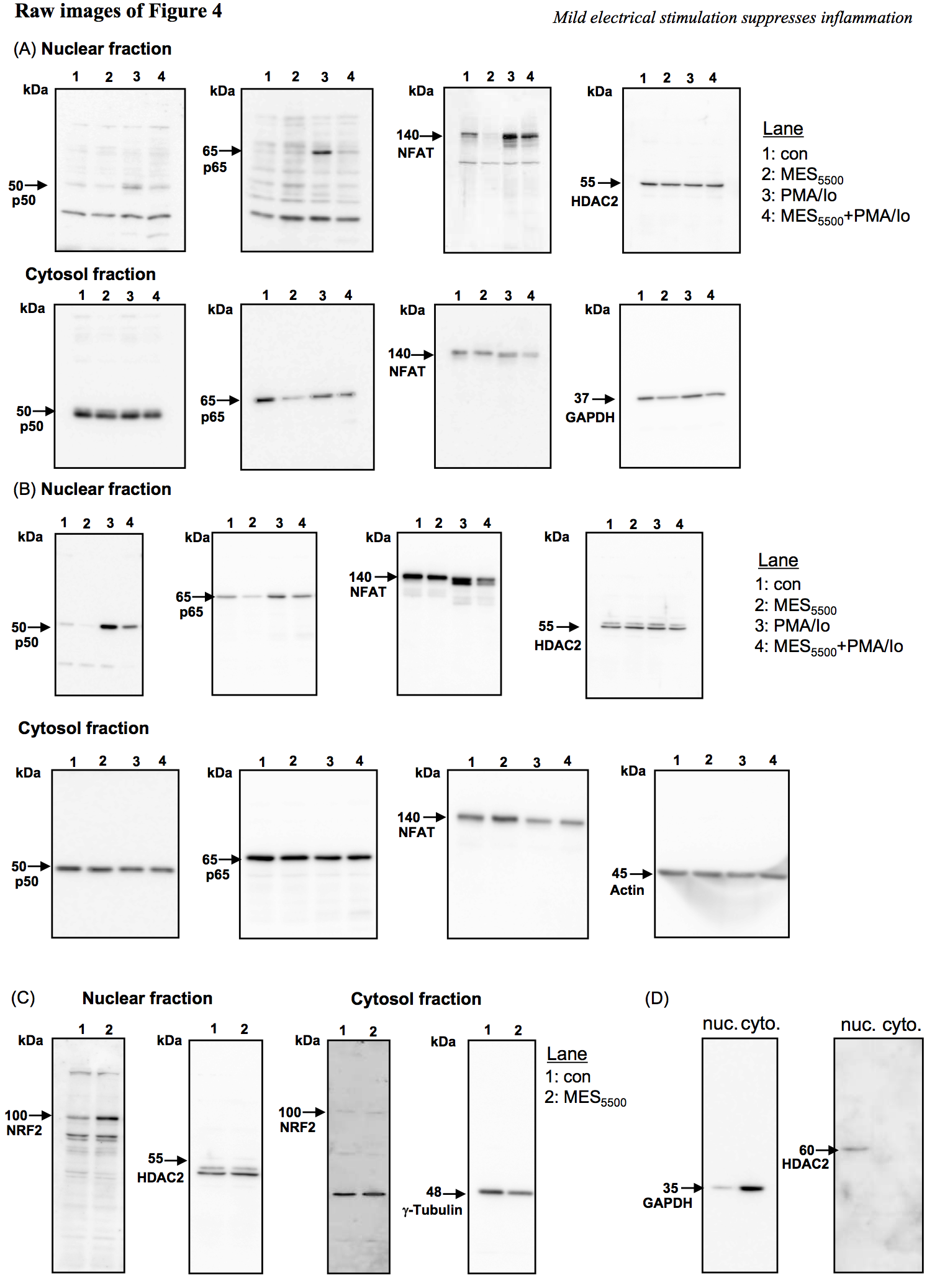

Supplement: S1 Raw images — (ZIP) [file pone.0234867.s010.zip › SI Raw images tiff/Raw images Fig 4.tiff]

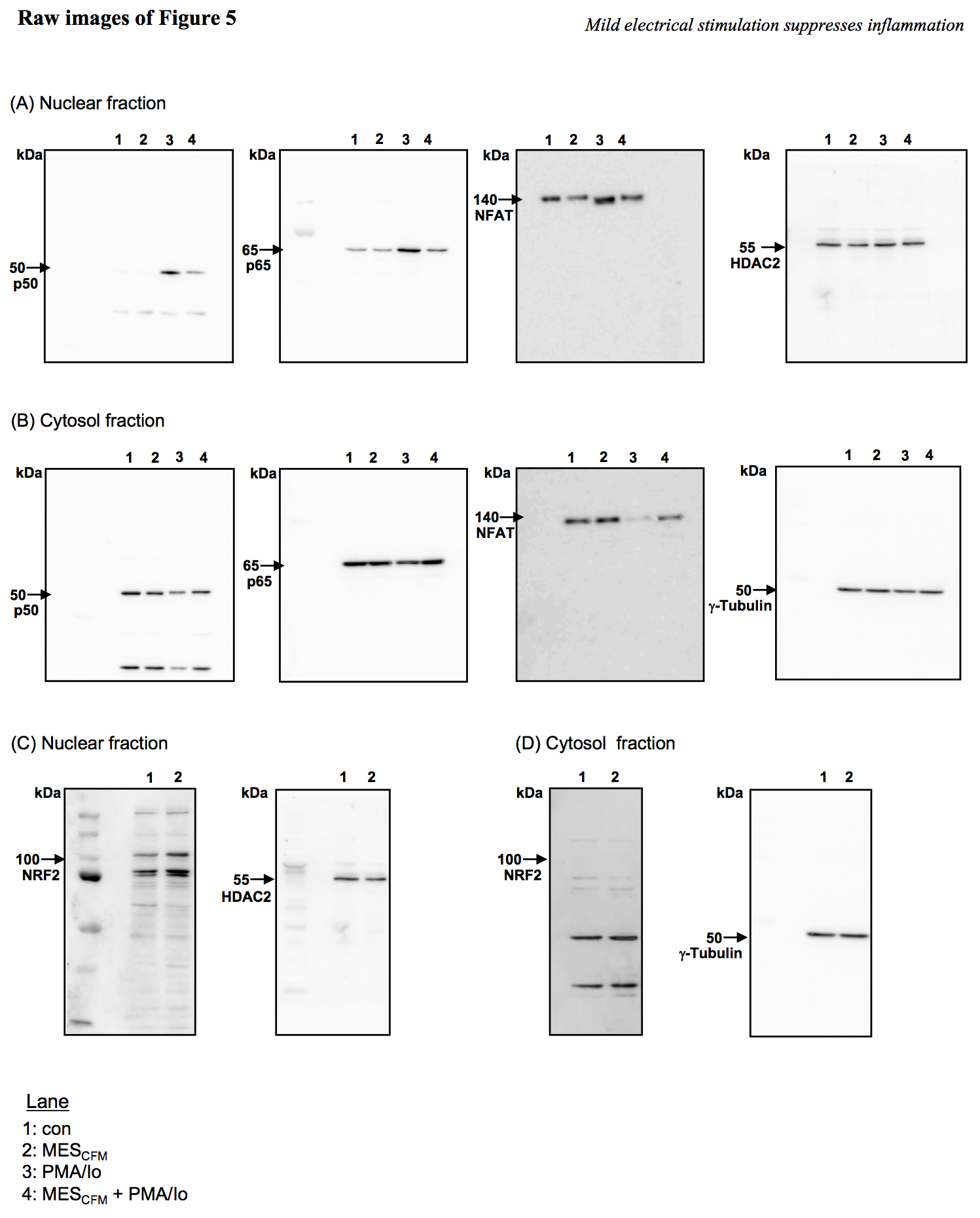

Supplement: S1 Raw images — (ZIP) [file pone.0234867.s010.zip › SI Raw images tiff/Raw images Fig 5.tiff]

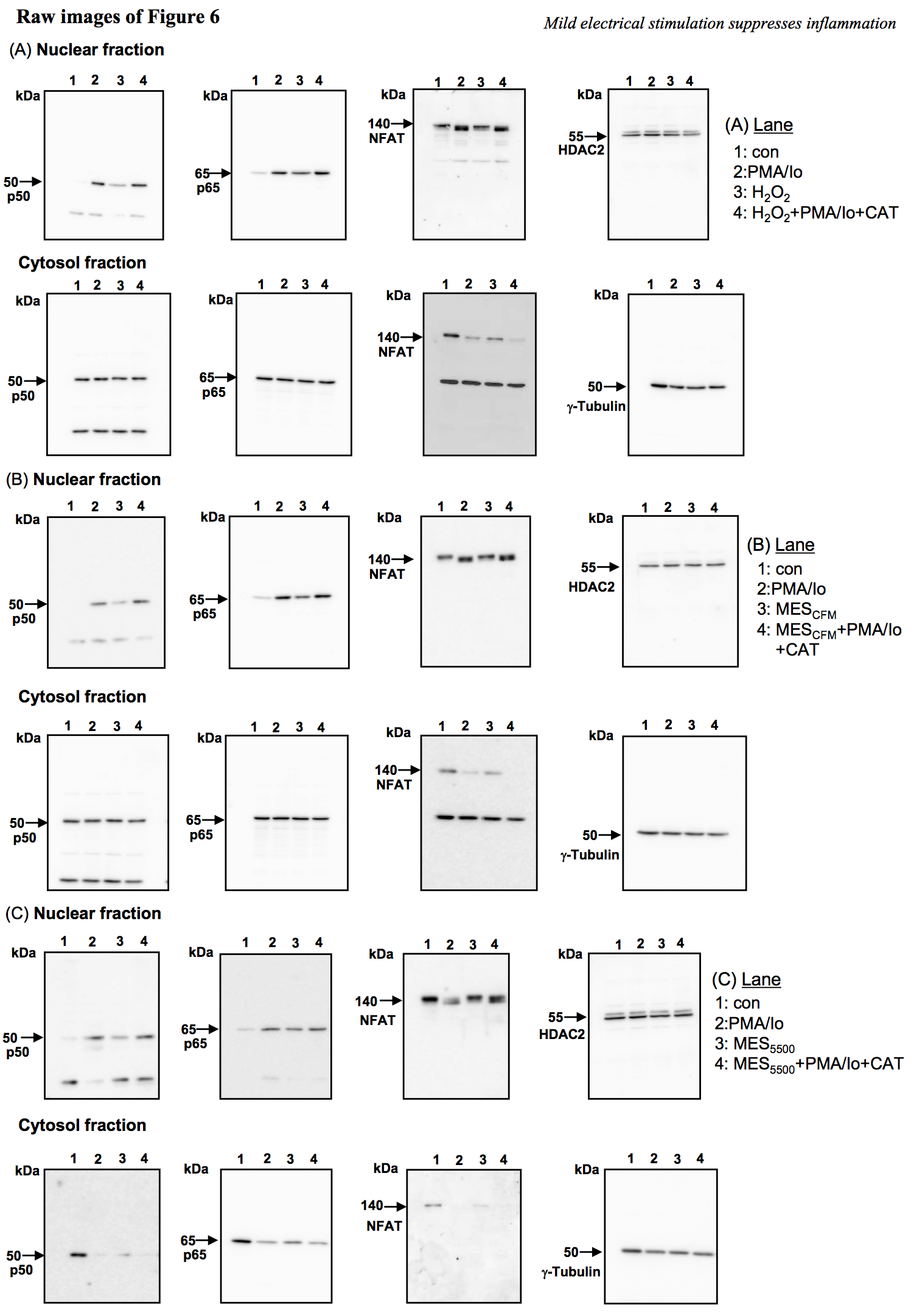

Supplement: S1 Raw images — (ZIP) [file pone.0234867.s010.zip › SI Raw images tiff/Raw images Fig 6.tiff]

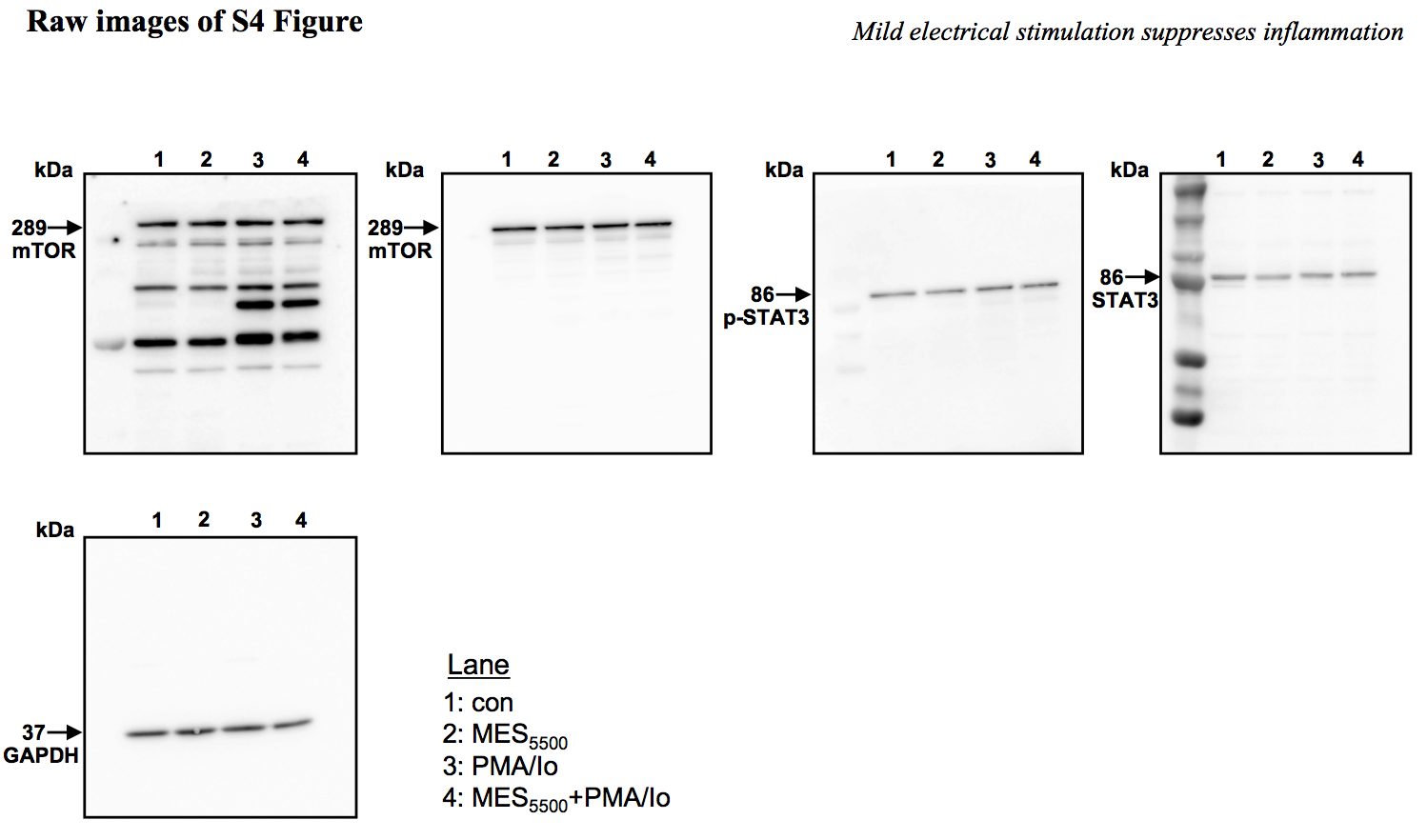

Supplement: S1 Raw images — (ZIP) [file pone.0234867.s010.zip › SI Raw images tiff/Raw images S4 Fig.tiff]

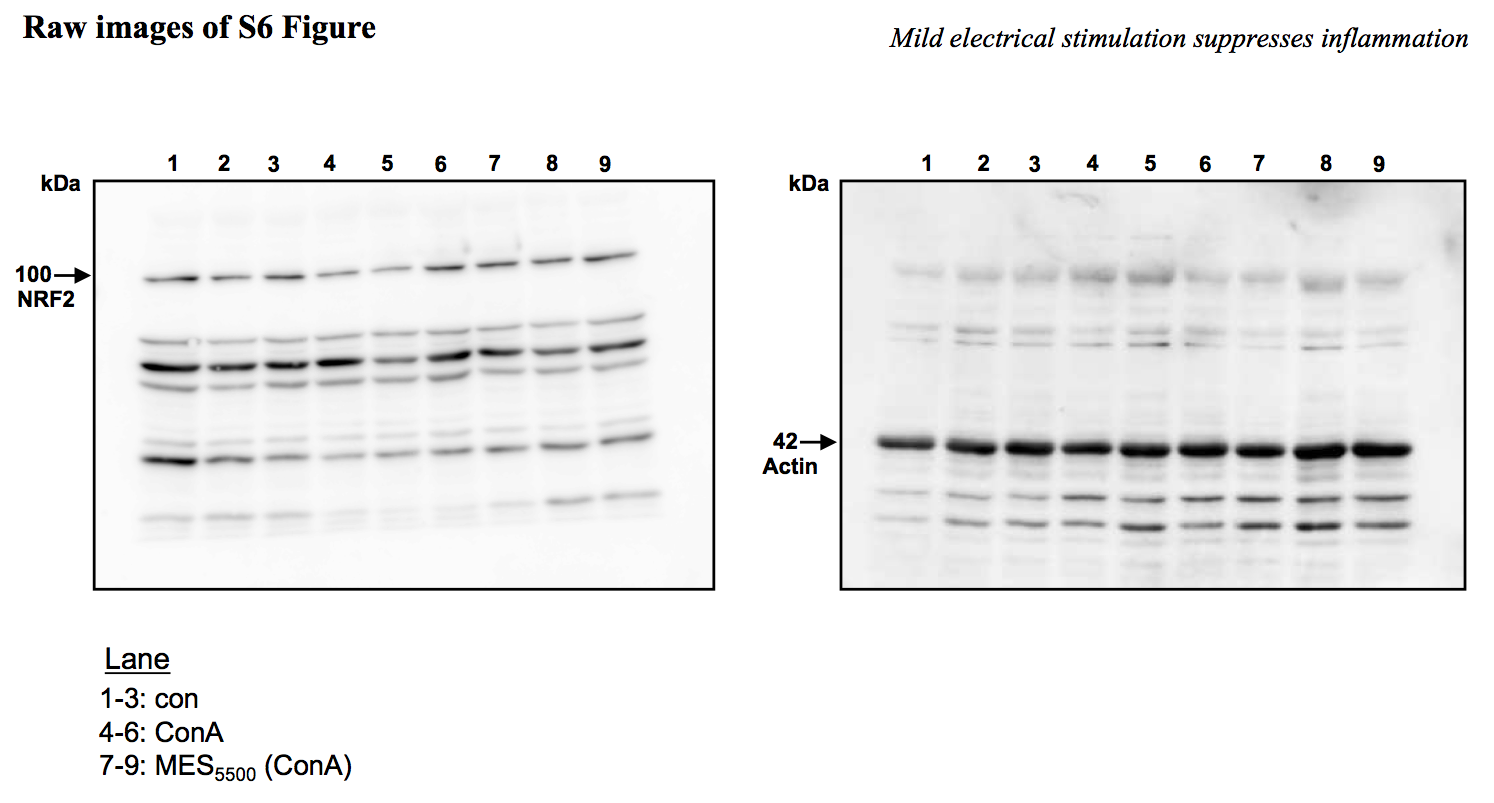

Supplement: S1 Raw images — (ZIP) [file pone.0234867.s010.zip › SI Raw images tiff/Raw images S6 Fig.tiff]
